# Oral Microbiota Composition and Its Association with Gastrointestinal and Developmental Abnormalities in Children with Autism Spectrum Disorder

**DOI:** 10.3390/microorganisms13081822

**Published:** 2025-08-04

**Authors:** Zuzanna Lewandowska-Pietruszka, Magdalena Figlerowicz, Katarzyna Mazur-Melewska

**Affiliations:** Department of Infectious Diseases and Child Neurology, Karol Marcinkowski University of Medical Sciences, 60-830 Poznan, Poland; lewandowska.pietruszka@gmail.com (Z.L.-P.); mfiglerowicz@ump.edu.pl (M.F.)

**Keywords:** autism spectrum disorder, oral microbiota, salivary cortisol, gut–brain axis, functional gastrointestinal disorder, diet, behavior

## Abstract

Autism Spectrum Disorder (ASD) is frequently accompanied by gastrointestinal disturbances, dietary selectivity, and altered stress responses, with growing evidence pointing to gut–brain axis involvement. While intestinal microbiota has been extensively studied, the role of the oral microbiota remains underexplored. This study investigates the associations between oral microbiota composition and behavioral, gastrointestinal, dietary, and neuroendocrine parameters in children with ASD. A total of 45 children aged 2–18 years comprised the study group. Data collection included oral swabs for 16S rRNA gene sequencing, salivary cortisol sampling, dietary records, and standardized behavioral assessments using the Vineland Adaptive Behavior Scale. A total of 363 microbial species across 11 phyla were identified. Significant correlations were observed between specific bacterial taxa and functional gastrointestinal disorders (FGIDs), dietary patterns, salivary cortisol rhythms, and functioning. Children with FGIDs, food selectivity, or macronutrient imbalances exhibited enriched pro-inflammatory taxa (e.g., *Selenomonas*, *Megasphaera*), whereas those with typical cortisol secretion or higher adaptive functioning showed greater microbial diversity and abundance of health-associated genera (e.g., *Bifidobacterium dentium*). These findings suggest that oral microbiota profiles may reflect systemic physiological and neurobehavioral traits in children with ASD. Further longitudinal studies are needed to clarify causal relationships and support the development of microbiota-targeted interventions.

## 1. Introduction

Autism Spectrum Disorder (ASD) is a heterogeneous neurodevelopmental condition characterized by early-onset and persistent deficits in social communication and social interaction, along with restricted, repetitive patterns of behavior, interests, or activities. According to the diagnostic criteria outlined in the International Statistical Classification of Diseases and Related Health Problems—10th Revision, ASD is defined by core abnormalities in communication and social interactions, and by stereotypical interests and behavior [1]. The World Health Organization estimates that up to 1 in 100 children worldwide presents with symptoms of autism; however, data coming from developing countries may be underestimated [2,3].

Recent advances in microbiome research have increasingly implicated the gut microbiota as a potential modulator of neurodevelopment and behavior, particularly in the context of ASD. Alterations in the composition and diversity of intestinal microbiota—referred to as dysbiosis—have been consistently observed in children with ASD compared to neurotypical controls [4,5]. These microbial imbalances are frequently accompanied by gastrointestinal symptoms, which occur in up to 70% of individuals with ASD, suggesting a strong gut–brain axis component in the disorder’s pathophysiology [6,7]. The gut–brain axis, a complex bidirectional signaling network involving neural, endocrine, and immune pathways, mediates interactions between the central nervous system and intestinal microbiota. Dysbiosis may contribute to ASD by affecting intestinal permeability, modulating the immune response, and altering neurotransmitter synthesis and availability [8,9]. Specifically, aberrant microbial metabolites such as short-chain fatty acids and lipopolysaccharides have been implicated in neuroinflammation and altered synaptic plasticity [7].

The available data on oral microbiota in ASD is scarce; however, the differences between neurodivergent individuals and typically developing children were previously observed. The abundance of specific genera in oral microbiota composition correlated with the intensity of core symptoms of ASD and intelligence quotients, as well as with the functioning of the patients in several domains, such as communication and social skills [7]. The oral microbiota composition differences were also observed in different neurological conditions, such as Alzheimer’s disease or Parkinson’s disease, further indicating the possible existence and importance of the oral–brain axis [10].

In addition to microbial and gastrointestinal factors, urine metabolite profiling has emerged as a promising non-invasive tool for identifying metabolic alterations associated with ASD [11,12]. Numerous studies have reported differences in urinary levels of metabolites related to amino acid metabolism, oxidative stress, neurotransmitter pathways, and mitochondrial function in individuals with ASD compared to neurotypical controls. These metabolic fingerprints reflect systemic physiological changes and may serve as potential biomarkers for early ASD diagnosis. Unlike behavioral assessments that are often subjective and age-dependent, urine-based assays offer objective, reproducible insights that can complement microbiota analysis and enhance classification accuracy. Integrating urine metabolomics with microbial and neuroendocrine profiling may therefore improve our understanding of ASD pathophysiology and aid in developing more precise diagnostic models.

Given these findings, microbiota-targeted interventions—including probiotics, prebiotics, dietary modifications, and fecal microbiota transplantation—have been proposed as potential adjunctive therapies for ASD. Preliminary studies have shown that such approaches may alleviate both gastrointestinal and behavioral symptoms [13]. However, further randomized controlled trials with standardized protocols are essential to validate their efficacy and elucidate the underlying mechanisms.

We hypothesized that children with ASD would present distinct oral microbiota profiles associated with both gastrointestinal and behavioral phenotypes, and that these microbial signatures would correlate with dietary patterns and cortisol rhythms.

The particular aims of this study were to (1) characterize the oral microbiota profile in children with ASD using 16S rRNA sequencing; (2) assess associations between oral microbial taxa and gastrointestinal symptoms, dietary intake, and food selectivity; (3) examine correlations between oral microbiota and adaptive functioning (VABS) and salivary cortisol secretion; and (4) identify potential microbial markers predictive of functional outcomes in ASD.

## 2. Materials and Methods

### 2.1. Participants

The participants in this study were recruited from the Department of Infectious Diseases and Child Neurology at the University of Medical Sciences in Poznań between January 2020 and January 2023.

The inclusion criteria were (1) diagnosis of ASD by a clinical team of psychologists and psychiatrists, in accordance with the ICD-10 diagnostic criteria [1,4,6]; (2) age between 2 and 18 years old.

The exclusion criteria were (1) organic gastrointestinal (GI) diseases, confirmed by a child gastroenterologist; (2) elimination diet due to organic diseases such as celiac disease, lactose intolerance, or allergies, confirmed by a specialist clinician; (3) diagnosed epilepsy, confirmed by a child neurologist; (4) pharmacotherapy in the last three months that could influence microbiota (such as antibiotics, probiotics, or acid suppression therapy); (5) stool transplantation at any time; (6) inability or unwillingness to complete all of the questionnaires and/or undergo sample collection in adherence to the methods and time frames planned for the research.

A total of 234 children’s guardians expressed interest in participating in this study. A total of 15 of them were excluded because of a GI disease, 29 because of an elimination diet, 18 due to epilepsy, 68 because of pharmacotherapy, and 59 due to inability or unwillingness to participate in all parts of the research.

After excluding ineligible patients, the research group consisted of 45 children. Informed consent was obtained from the legal guardians, permitting the collection of samples and clinical data.

### 2.2. The Medical History Analysis

Guardians, along with their children, completed a personal questionnaire that included inquiries about the diagnosis according to ICD-10, age, gender, mode of delivery (vaginal versus Cesarean section), and comorbidities. Additionally, caregivers maintained an estimated seven-day food diary detailing the child’s food intake (breakfast, second breakfast, lunch, afternoon snack, and dinner) along with snacks and fluids, including brand names. Each participant was given a standardized form to fill in manually. An estimated number of portions and meal times was provided by the caregivers [14,15]. Based on the diary data, calorie and fluid intake were calculated, as well as the food composition regarding fat, carbohydrates, and proteins. Patients were categorized into groups based on dietary intake: low-calorie diet (LCD; not exceeding the maximum normal number of calories for the patient’s sex and age) or high-calorie diet (HCD; exceeding normal levels), as well as protein-based diet (PBD; more than 20–30% of daily energy) and carbohydrate-based diet (ChBD). Furthermore, children were assessed using a questionnaire for symptoms of functional gastrointestinal disorders according to the Rome IV Criteria. The VABS Third Edition was utilized to assess the patients’ functioning, with scores evaluated across the main domains of communication, daily living skills, socialization, and motor skills. Subdomains including receptive, expressive, and written communication; daily living skills; socialization; and gross and fine motor skills were also assessed, allowing for the identification of groups based on functioning levels.

### 2.3. Sample Collection and Storage

The oral swabs were taken individually by the first author. The samples were collected from children on an empty stomach in an indoor environment. Immediately prior to sampling, the patient’s body temperature was measured, and a history of contact with sick individuals was collected. The oral swab was obtained by moving a flocked swab around the tongue, buccal area, and hard palate for at least 25 s, after which it was placed in a swab tube and frozen at −80 degrees Celsius within one hour. Additionally, saliva samples were collected from each child three times on the same day using a Salivette^®^ Cortisol tube (SARSTEDT AG & Co. KG, Nümbrecht, Germany) with a synthetic swab, at 8 a.m., 2 p.m., and 8 p.m. The swab was placed in the child’s oral cavity, and the child was instructed to chew for approximately 60 s. The tube was then stored in a freezer at −20 degrees Celsius until further analysis. All samples, including oral swabs and cortisol samples, were anonymized using a generated code number to maintain participant confidentiality and reduce potential bias.

### 2.4. Microbiome Assessment

Genomic DNA isolation was performed by trained laboratory personnel using an Auto-Pure Mini (Allsheng, Hangzhou, China) device with MagnifiQ (A&A Biotechnology, Gdansk, Poland) reagents. The preparation of DNA libraries was achieved through PCR amplification of the V3–V4 fragment of 16S rRNA. Subsequent microbiome analysis was conducted using Next Generation Sequencing of bacterial 16S RNA with MiSeq (Illumina, San Diego, CA, USA) sequencers. All DNA samples included in this study were of sufficient quality to proceed with downstream 16S rRNA gene sequencing and analysis. None of the samples were excluded due to low concentration or degradation, and all underwent identical processing through the bioinformatics pipeline. Sequence quality control was performed using the DADA2 algorithm implemented within QIIME 2. The denoising parameters were set to truncate both forward and reverse reads at 260 base pairs, trim the first 10 bases of forward and 20 bases of reverse reads, and allow a maximum of two expected errors per read. Additionally, reads were truncated at the first base with a quality score below 2. Chimeric sequences were identified and removed using the consensus method, and only features with a total frequency of at least 10 were retained for further analysis. After denoising and abundance filtering, taxonomic filtering was conducted to refine the dataset by excluding sequences of non-bacterial origin. Specifically, features assigned to mitochondria and chloroplasts were removed, followed by the exclusion of all sequences assigned to the domains Archaea and Eukaryota. The taxonomic classification required for this filtering was carried out using the QIIME 2 classify–consensus–blast method, referencing the SILVA 138-99 database for both taxonomy and sequence alignment.

The examination was performed at the certified research laboratory of the SANPROBI Research and Development Centre in Szczecin, Poland, under the supervision of specialized scientists from the Pomeranian Medical University in Szczecin.

### 2.5. Cortisol Analysis

For cortisol analysis, the Salivette^®^ Cortisol tubes were thawed and centrifuged for 2 min at 1000× *g* to recover the collected saliva. A solid-phase competitive Enzyme-Linked Immunosorbent Assay (ELISA) was conducted to assess cortisol levels, utilizing a commercial Invitrogen Human Cortisol Competitive ELISA Kit (Thermo Fisher Scientific, Waltham, MA, USA) The examination was performed in the laboratory affiliated with the Department of Infectious Diseases and Child Neurology in Poznan.

### 2.6. Statistics and Biostatistics

Data analysis was conducted by a qualified biostatistician using R version 4.3.2 (31 October 2023 ucrt). Correlations between different microbiota compositions and modifying factors were analyzed using Spearman’s and Winsorized Pearson’s correlation, while Pearson’s correlation was used to explore multivariate aspects of microbiota composition. The choice between Pearson and Spearman correlation tests was based on the assessment of bivariate normality, evaluated using the Shapiro–Wilk test. A *p*-value of ≤0.05 was considered statistically significant.

## 3. Results

### 3.1. Oral Microbiota Sequencing

Oral microbiota analysis based on salivary DNA samples revealed the presence of microbial communities spanning 11 phyla, 16 classes, 42 orders, 83 families, 223 genera, and 363 species. The microbiota composition varied greatly between the participants; however, some trends were visible within the majority of the group. Most of the children were most abundant in *Bacilli*, especially in the genera *Streptococcus* and *Gemella*, and *Actinobacteria,* especially *Rothia* and *Actinomyces*. There is no consensus on the normal *Firmicutes* to *Bacteroidetes* ratio; however, in comparison to available data [16], it was visibly low in all participants (mean 0.21 ± 0.15). Subsequent analyses investigated associations between microbiota diversity on different taxonomic levels and various clinical, dietary, and physiological characteristics of the study population, including the occurrence of FGIDs, diet and food selectivity, and functioning of the participants in several domains.

### 3.2. The Structure of the Group

This study included 45 participants, with a predominance of boys (38 children, 84%) and a mean age of 7.9 ± 3.35 years. A total of 12 children (45%) belonged to the youngest age group (between 2 and 6 years old), 28 of them (62%) to the middle age group (6 to 14 years old), and 5 of them (11%) to the oldest age group (15 to 18 years old). Vaginal delivery was the most common mode of birth (31 cases, 69%). The most frequently reported gastrointestinal symptom was food selectivity (16 children, 36%).

In terms of dietary analysis, the average protein intake was 90.05 g/day (3.81 g/kg BW), carbohydrate intake was 251.02 g/day (51.41% of total energy), and fat intake was 79.51 g/day (3.3 g/kg BW), with unsaturated fats accounting for 59.89% of total fat intake. The detailed participants’ characteristics are included in Table 1.

**Figure 1:**
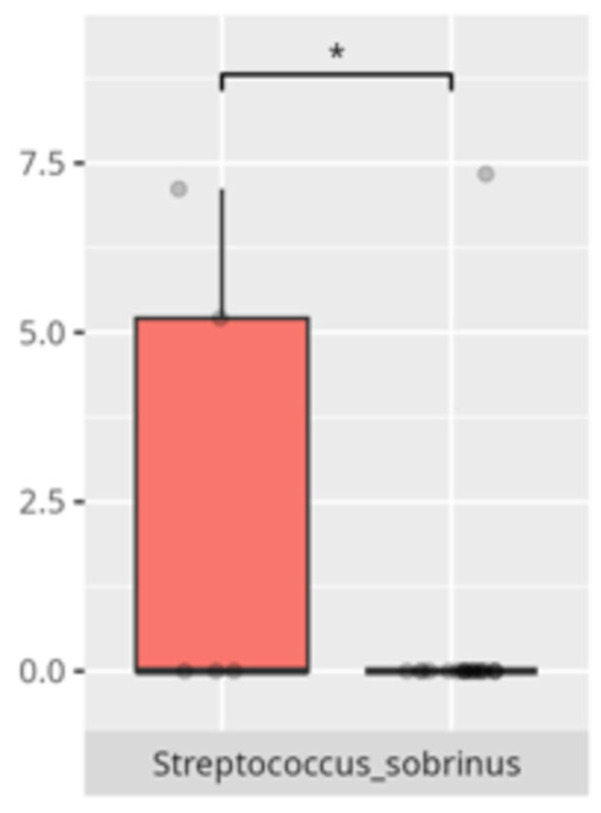
Statistically significant differences between two different sex groups (red—female, blue—male; * *p* ≤ 0.05).

**Figure 2:**
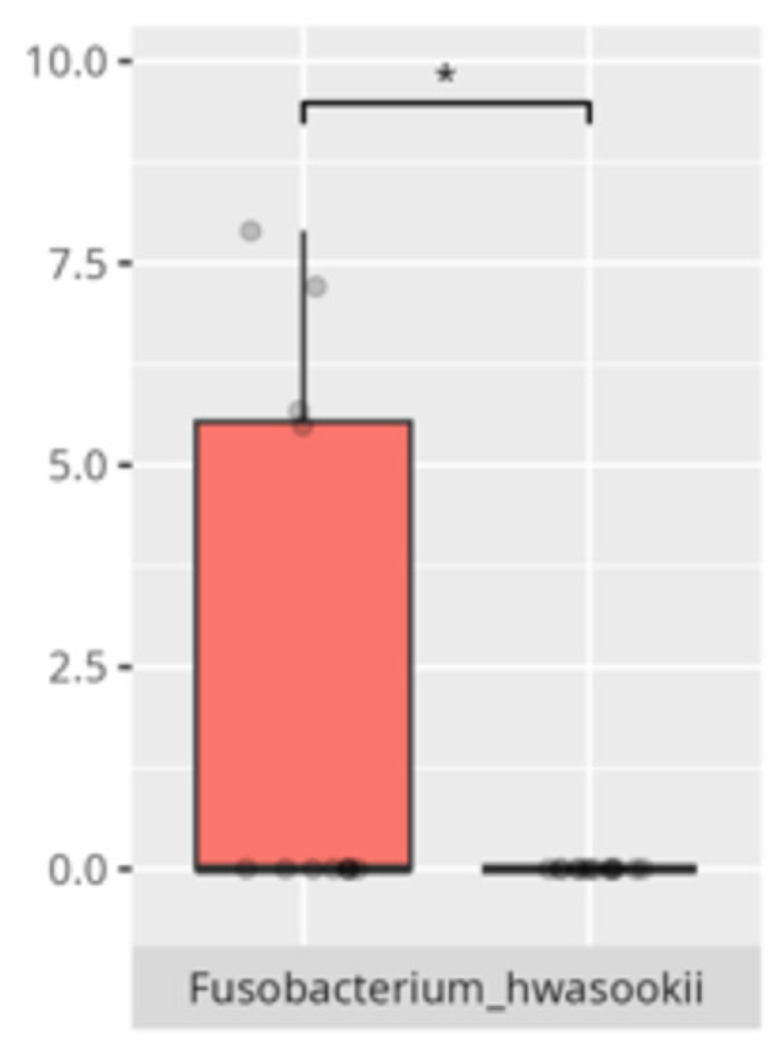
Statistically significant differences between two different groups according to the mode of delivery (red—Cesarean section, blue—vaginal delivery; * *p* ≤ 0.05).

**Figure 3:**
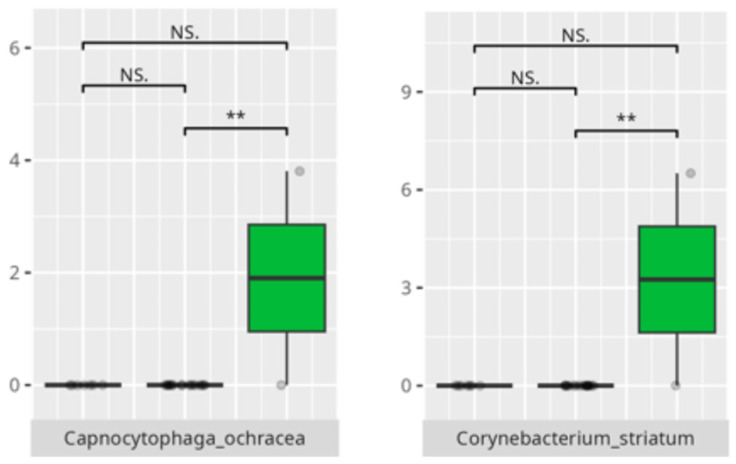
Statistically significant differences between different age groups (1st column—youngest children, 2nd column—oldest children, and 3rd column—middle children; ** *p* ≤ 0.01; NS.—not significant).

Significant differences were observed in the composition of the oral microbiota across various participant subgroups. The differences are listed in Table 2 and visualized in Figure 1, Figure 2 and Figure 3.

### 3.3. Functional Gastrointestinal Disorders

Forty percent of participants (*n* = 18) met the diagnostic criteria for at least one FGID. The most frequently reported conditions included functional diarrhea (*n* = 9; 20%), constipation (*n* = 8; 18%), and bloating (*n* = 7; 16%). Among children with FGIDs, 4 (33%) were in the youngest age group, 11 (39%) in the middle age group, and 3 (60%) in the oldest group. The majority were boys (*n* = 16; 89%).

**Figure 4:**
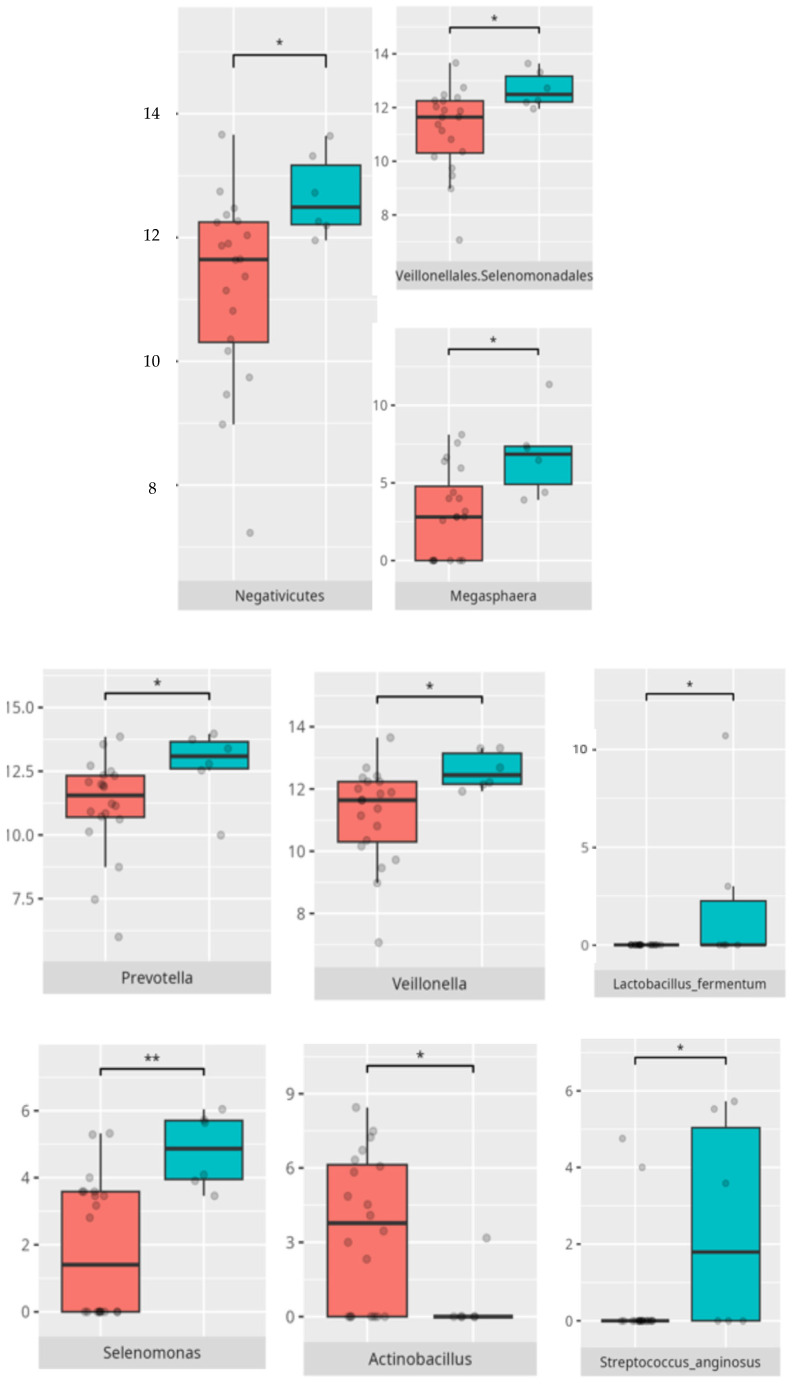
Statistically important differences between groups—diarrhea (red—no diarrhea, blue—diarrhea; *—*p* ≤ 0.05, **—*p* ≤ 0.01).

**Figure 5:**
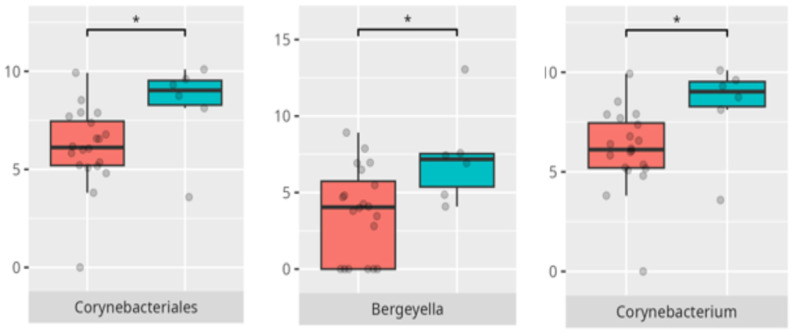
Statistically important differences between groups—bloating (red—no bloating, blue—bloating; *—*p* ≤ 0.05,).

**Figure 6:**
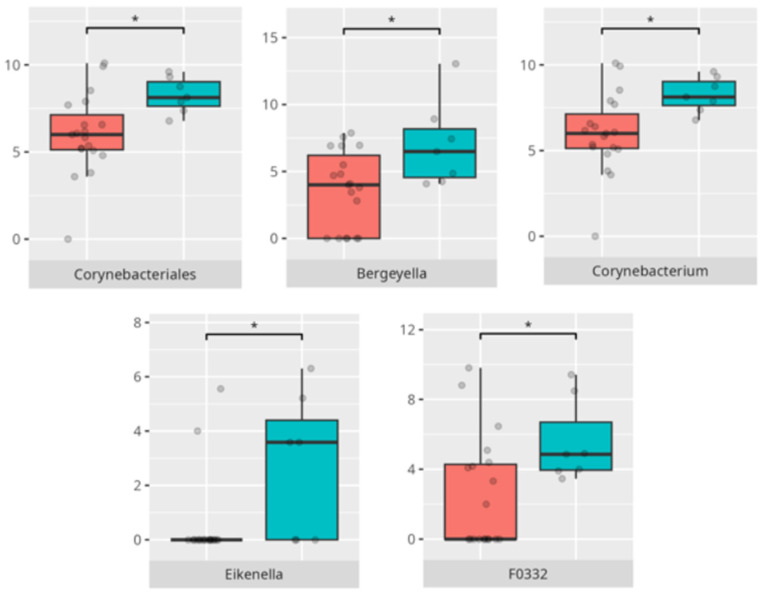
Statistically important differences between groups—constipation (red—no constipation, blue—constipation; * *p* ≤ 0.05).

**Figure 7:**
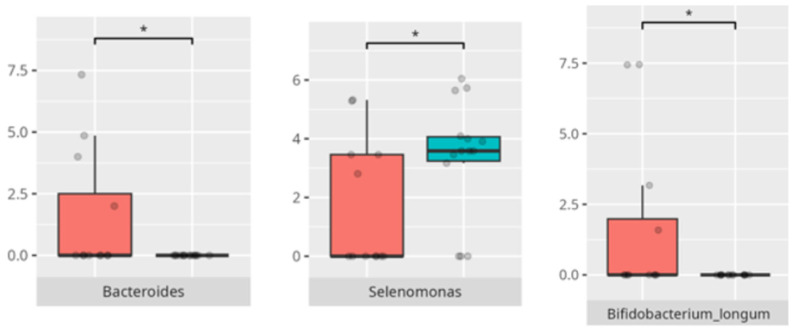
Statistically important differences between groups—functional gastrointestinal disorders (FGIDs) (red—no FGID, blue—FGID; * *p* ≤ 0.05).

Significant differences were observed in the composition of the oral microbiota depending on the occurrence of FGIDs. The differences are listed in Table 2 and visualized in Figure 4, Figure 5, Figure 6 and Figure 7.

### 3.4. Diet and Food Selectivity

The mean daily protein intake among participants was 90.05 ± 31.06 g, corresponding to 3.81 ± 1.71 g/kg of body weight. The average daily carbohydrate intake was 251.02 ± 62.66 g, with simple sugars comprising 41.31 ± 7.82% of total carbohydrate consumption. Carbohydrates contributed 51.41 ± 9.31% to total daily energy intake. Fat intake averaged 79.51 ± 31.32 g/day (3.3 ± 1.7 g/kg), with unsaturated fats accounting for 59.89 ± 5.30% of total fat intake.

Most children (*n* = 31; 69%) followed a protein-based diet (PBD), while approximately half (*n* = 23; 51%) reported adherence to a low-calorie diet (LCD). A carbohydrate-based diet (ChBD) was declared by the parents of 50% (*n* = 6) of the youngest participants, 25% (*n* = 7) of those in the middle age group, and by only 1 participant in the oldest group. Most children on ChBDs (*n* = 12; 86%) were boys, and a majority (*n* = 8; 57%) did not present symptoms of FGIDs. Caregivers frequently reported protein intake levels exceeding dietary recommendations for healthy children. Despite these elevated values, no statistically significant correlation was found between protein intake and the abundance of any specific bacterial genus.

A high-calorie diet (HCD) was reported in all participants from the youngest age group (*n* = 12), 32% (*n* = 9) of those in the middle age group, and only 1 child from the oldest group. The majority of these children (*n* = 19; 86%) were boys, and most (*n* = 14; 63%) did not present with FGID symptoms.

Food selectivity (FS) was observed in 36% (*n* = 16) of the participants, with a predominance of boys (*n* = 13; 81%), primarily within the middle age group (*n* = 10; 63%). Of these, 25% (*n* = 4) also exhibited FGID symptoms. Among children with FS, 69% (*n* = 11) followed an HCD. The proportion following protein-based and ChBDs was approximately equal.

**Figure 8:**
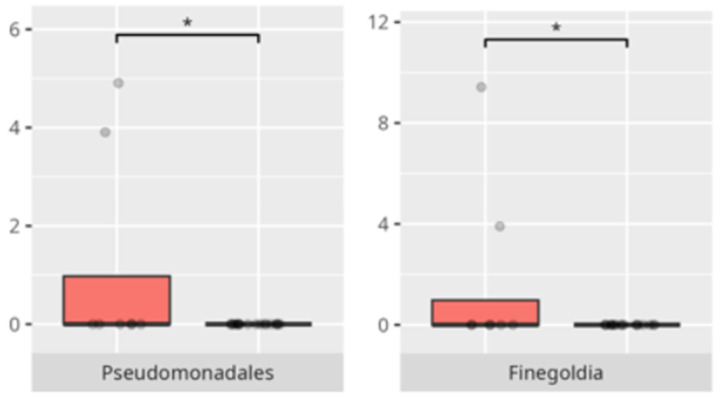
Statistically important differences between groups—protein intake (red—low-protein diet, blue—high-protein diet; * *p* ≤ 0.05).

**Figure 9:**
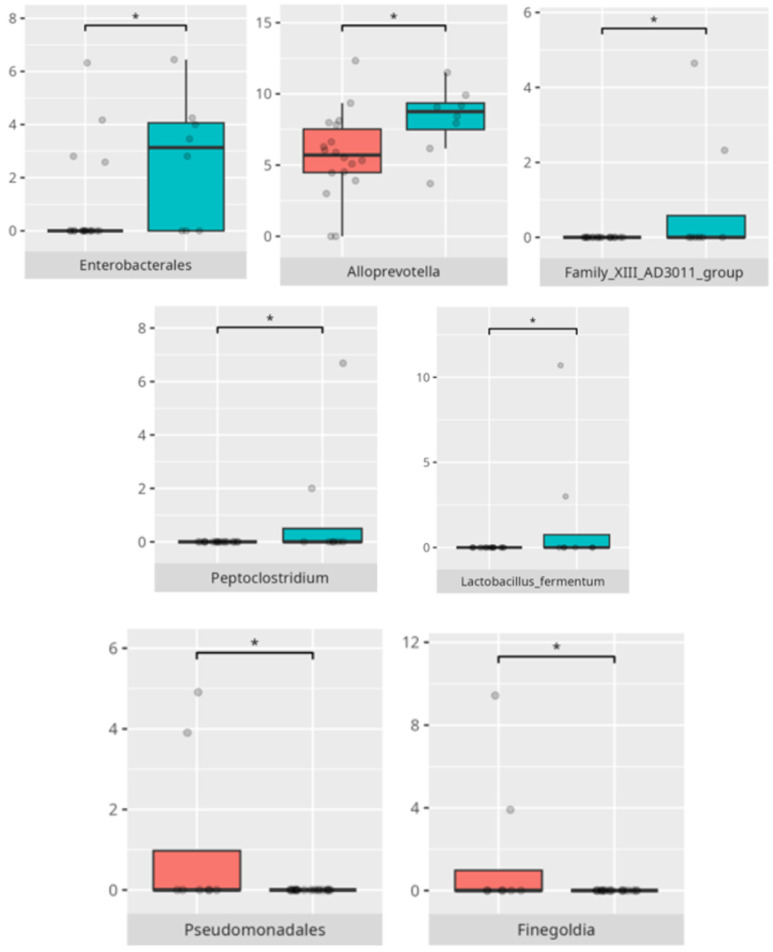
Statistically important differences between groups—food selectivity (red—no food selectivity, blue—food selectivity; * *p* ≤ 0.05).

The significant differences in the microbiota composition depending on diet and food selectivity are listed in Table 2 and visualized in Figure 8 and Figure 9.

At the genus level, the mean fluid intake positively correlated with the abundance of *Eubacterium yurii* (r = 0.55). The ratio of saturated to total fat intake was positively associated with *Alloscardovia* (r = 0.69), *Butyrivibrio* (r = 0.57), and *Mobiluncus* (r = 0.81).

At the species level, the saturated fat to total fat intake ratio was positively correlated with the abundance of *Bifidobacterium dentium* (r = 0.54) and *Mobiluncus curtisii* (r = 0.81). Intake of complex carbohydrates negatively correlated with *Streptococcus mutans* abundance (r = −0.53). Similarly, the ratio of energy derived from carbohydrates to the mean total carbohydrate intake was inversely associated with *Streptococcus mutans* abundance (r = −0.53).

All correlations are listed in Table 3.

### 3.5. Vineland Adaptive Behavioral Scale Results

All patients were assessed for their functioning in four main domains: communication, daily living skills, socialization, and motor skills. Furthermore, their adaptive behavior in each domain was further analyzed in more detailed subdomains. We compared three groups of children (scoring low, average, or high in each domain and subdomain) with the abundance of microbiota in the class, order, genus, and species levels, listing the statistically significant differences. Furthermore, we looked for the positive or negative correlations between the oral microbiota composition, diet, age, weight, salivary cortisol levels, and V-score obtained using the VABS.

#### 3.5.1. Domain: Communication

Thirty-one children scored within the low or moderately low range in the communication domain. Only four participants obtained average scores, while ten achieved high or moderately high results.

**Figure 10:**
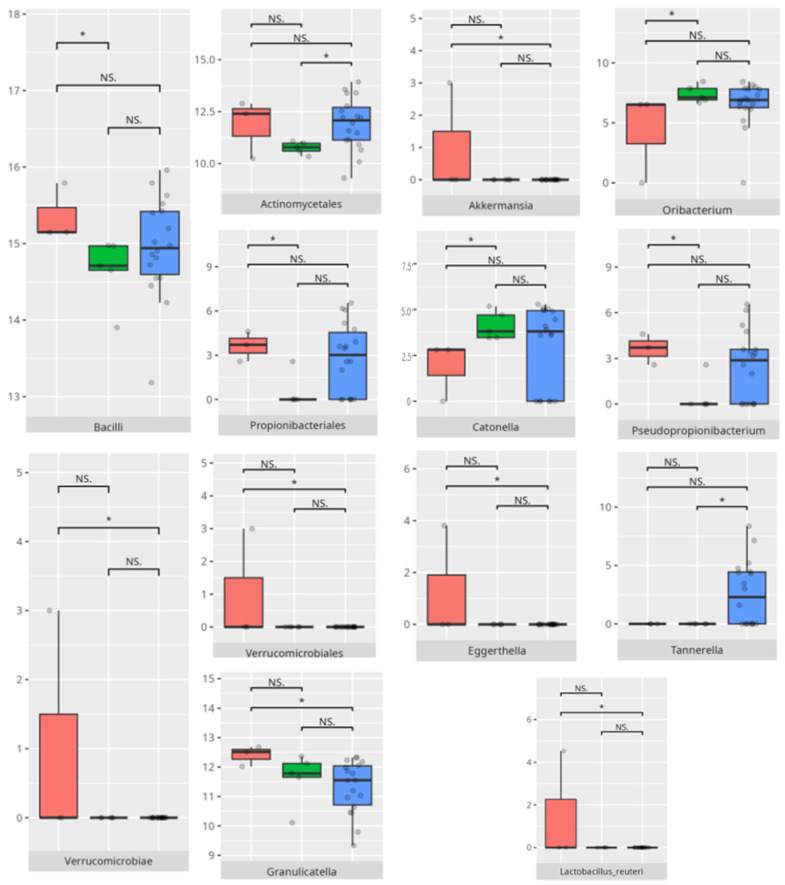
Statistically important differences between groups—total communication (red—average, green—low, and blue—high; * *p* ≤ 0.05; NS.—not significant).

The significant differences in the microbiota composition are listed in Table 2 and visualized in Figure 10.

##### Subdomain: Receptive Communication

Ten children scored within the high or moderately high range in the receptive communication subdomain, while 27 received low or moderately low scores. Among the high scorers, seven were in the middle age group and three were older boys. Three patients presented with FGIDs, and three exhibited FS. Most of them followed a PBD (*n* = 9) and an LCD (*n* = 8).

The lower-scoring group included 11 younger, 15 middle-aged, and 1 older child. Nine of these participants had alternative diagnoses. Ten had FGIDs, and eleven showed signs of FS. The majority adhered to a protein-based (*n* = 17) and high-calorie (*n* = 15) diet.

**Figure 11:**
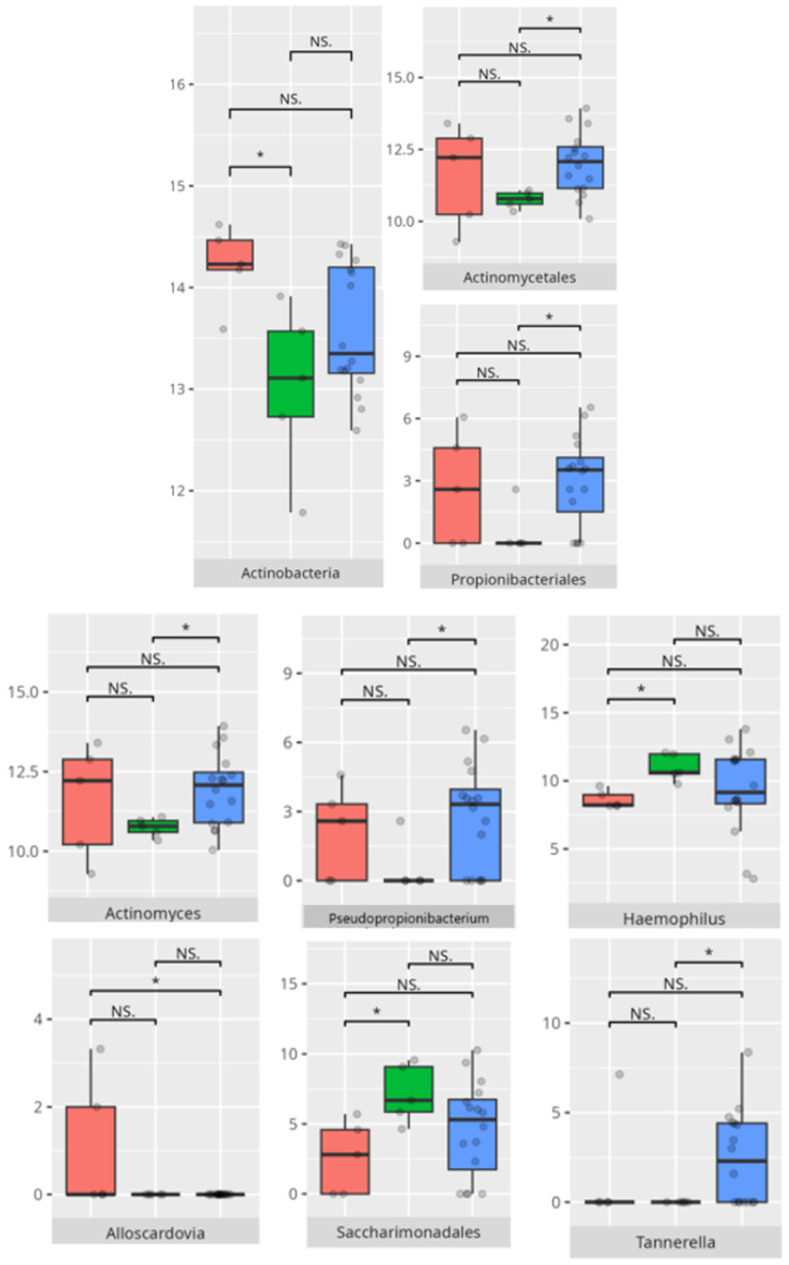
Statistically important differences between groups—receptive communication (red—average, green—low, and blue—high; * *p* ≤ 0.05; NS.—not significant).

The significant differences in the microbiota composition are listed in Table 2 and visualized in Figure 11.

##### Subdomain: Expressive Communication

Ten children demonstrated high or moderately high scores in the expressive communication subdomain, while twenty-four were categorized as having low or moderately low scores. Among the high-scoring group, eight children belonged to the middle age category, and two were older. Two children exhibited symptoms of FGIDs, and three of FS. The majority followed a protein-based (*n* = 9) and low-calorie (*n* = 8) diet. In contrast, the low-scoring group consisted mostly of boys (*n* = 20), including eight from the youngest age group, fourteen from the middle group, and two from the oldest group. Nine children in this group presented with FGIDs, and eleven demonstrated FS. Most followed a PBD (*n* = 14), and half also adhered to an LCD dietary pattern.

**Figure 12:**
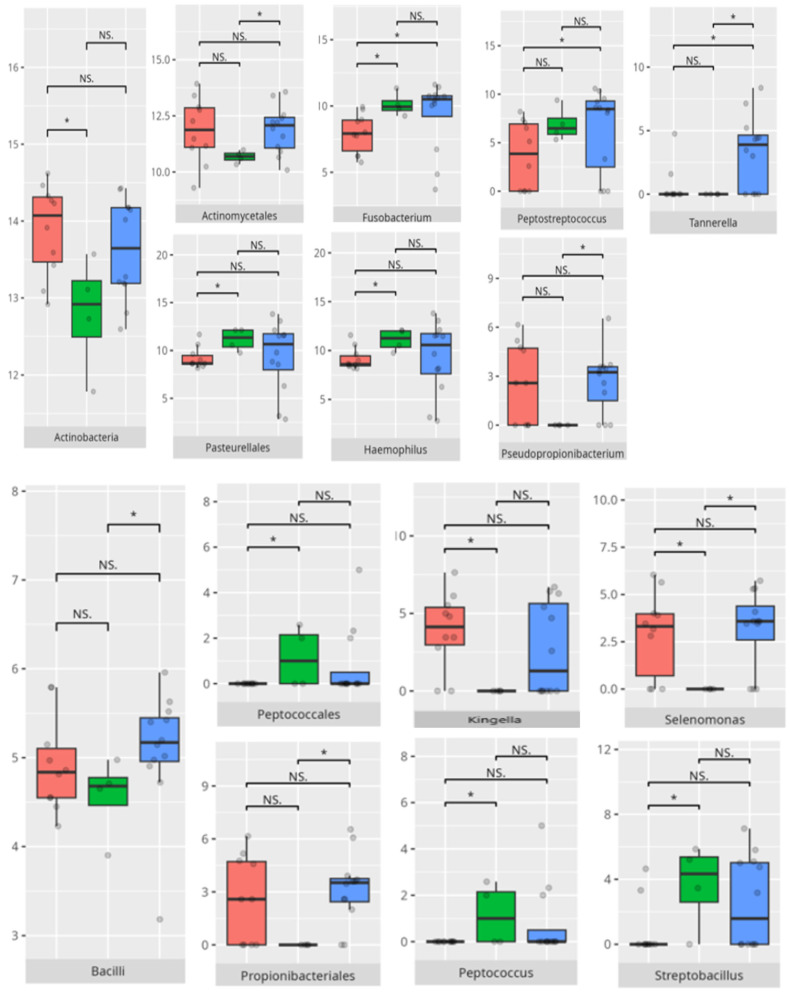
Statistically important differences between groups—expressive communication (red—average, green—low, and blue—high; * *p* ≤ 0.05; NS.—not significant).

The significant differences in the microbiota composition are listed in Table 2 and visualized in Figure 12.

##### Subdomain: Writing Skills

In the writing skills subdomain, five children achieved high or moderately high scores, whereas thirty-two were classified as having low or moderately low performance. All the children in the high-scoring group were boys from the middle age group. Two of them exhibited symptoms of FGIDs, and one of FS. Three patients followed a PBD, and all adhered to an LCD regimen.

All participants in the low-scoring group, of whom sixteen were boys, also belonged to the middle age category. Nine presented with FGIDs, and eight had FS. Fifteen children in this group followed a PBD, and twelve reported an LCD.

**Figure 13:**
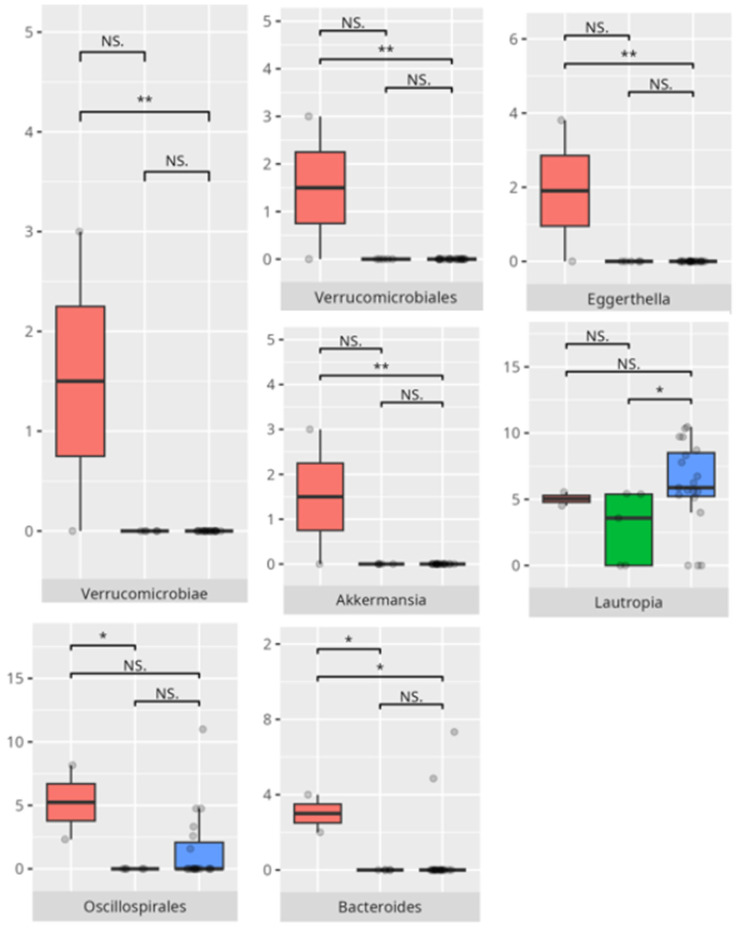
Statistically important differences between groups—writing skills (red—average, green—low, and blue—high; *—*p* ≤ 0.05, **—*p* ≤ 0.01; NS.—not significant).

The significant differences in microbiota composition are listed in Table 2 and visualized in Figure 13.

#### 3.5.2. Domain: Daily Living Skills

A total of 27 children exhibited low or moderately low scores in the daily living skills domain, whereas 10 demonstrated average performance and 8 achieved high or moderately high results. Children with lower daily living skills scores had a greater relative abundance of Bacilli compared to those with higher scores.

**Figure 14:**
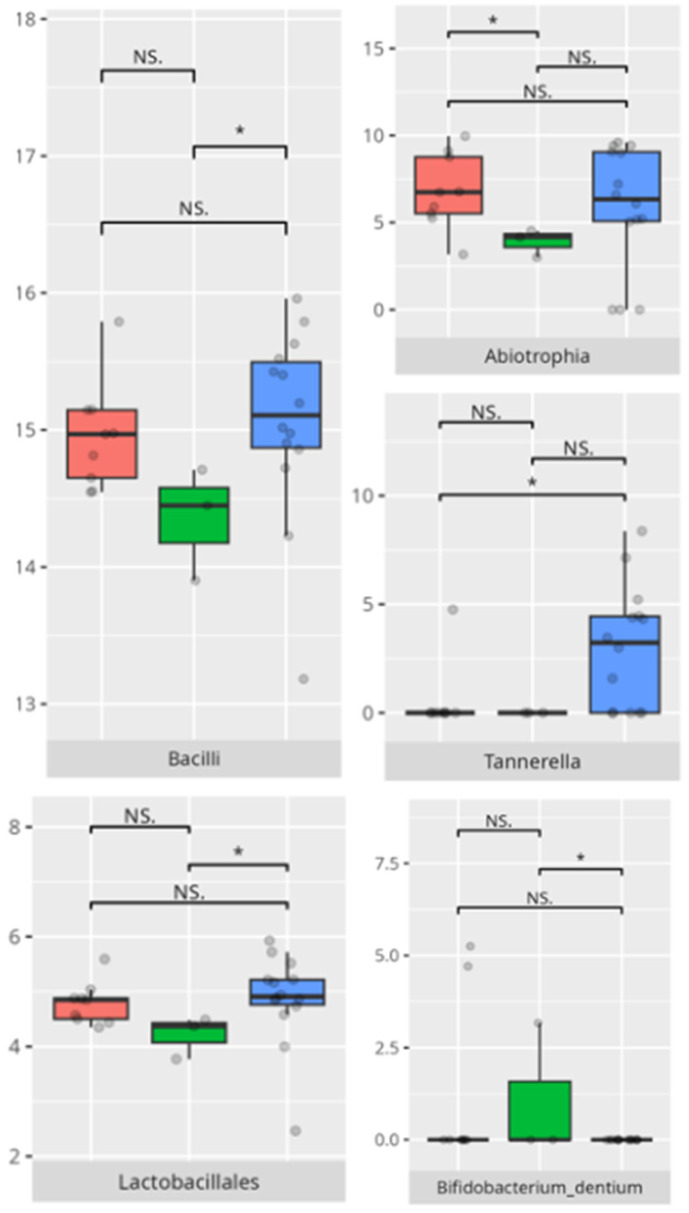
Statistically important differences between groups—daily living skills: total (red—average, green—low, and blue—high; * *p* ≤ 0.05; NS.—not significant).

The significant differences in the microbiota composition are listed in Table 2 and visualized in Figure 14.

##### Subdomain: Personal Skills

A total of 8 participants achieved high or moderately high scores in the personal skills subdomain, while 27 obtained low or moderately low scores. All participants with high scores were male—six from the middle age group and two from the older group. Two of them presented FGIDs, and three exhibited FS. Most followed a protein-based (*n* = 6) and low-calorie (*n* = 7) diet. Among the lower-scoring participants, the majority were boys (*n* = 20). This group included 8 younger, 14 middle-aged, and 2 older children. FGIDs were observed in nine participants, and FS in 11. Most adhered to a PBD (*n* = 14), and 11 also consumed an LCD.

**Figure 15:**
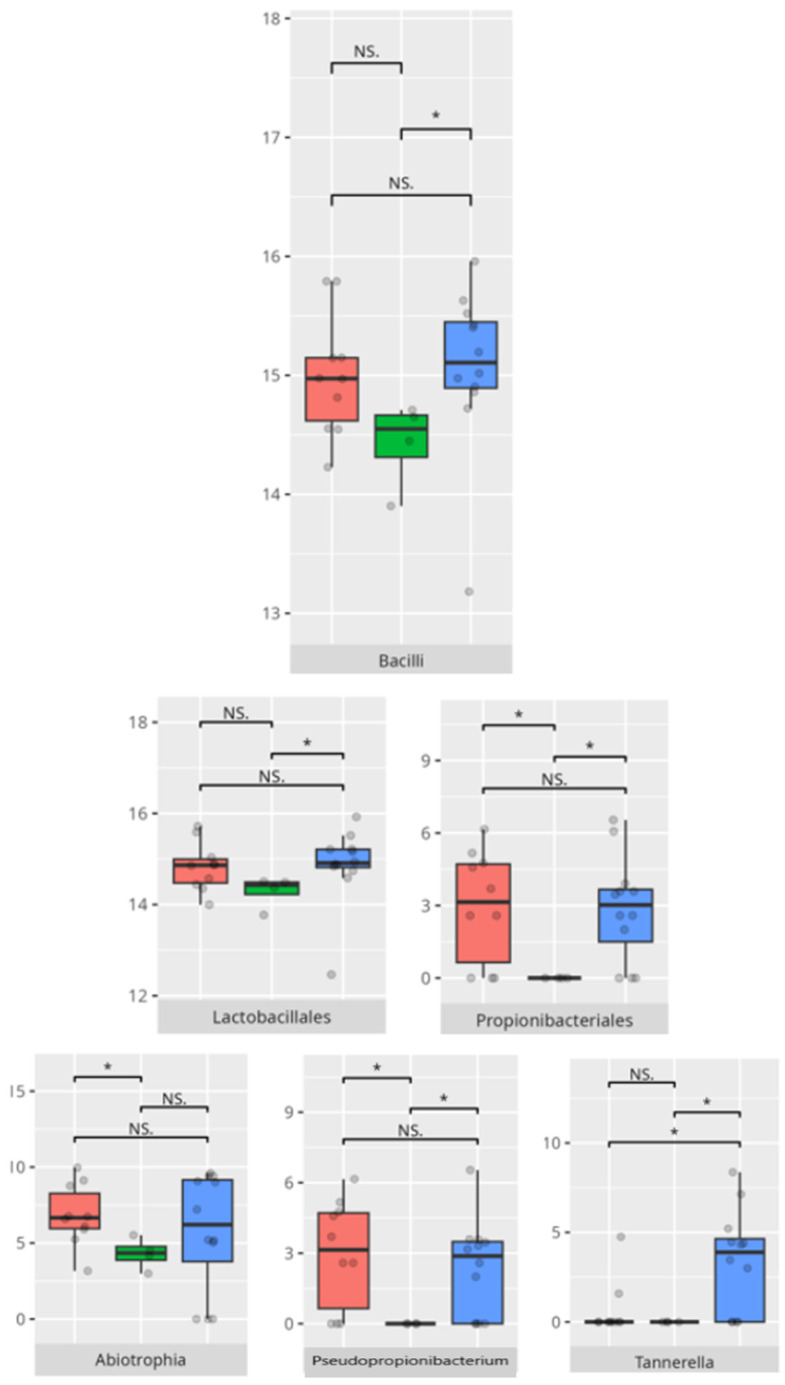
Statistically important differences between groups—daily living skills: personal skills (red—average, green—low, and blue—high; * *p* ≤ 0.05; NS.—not significant).

The significant differences in the microbiota composition are listed in Table 2 and visualized in Figure 15.

A significant negative correlation was observed between personal skills V-score and *Tannerella* abundance (r = −0.53) (Table 3).

##### Subdomain: Domestic Skills

A total of 7 male participants demonstrated high or moderately high proficiency in the domestic skills subdomain, while 27 individuals exhibited low or moderately low functional outcomes. Among those with stronger abilities, five belonged to the middle-aged group and two to the older group. Two children presented with FGIDs, and two with FS. Most adhered to a protein-based (*n* = 5) and low-calorie (*n* = 7) dietary regimen. Within the group displaying diminished capabilities in domestic functioning, 7 patients were younger children, 17 were in the middle age group, and 3 were older. FGIDs were identified in 11 cases, and 13 children exhibited FS. The majority followed a protein-rich diet (*n* = 18), with 14 adhering to an HCD dietary pattern.

**Figure 16:**
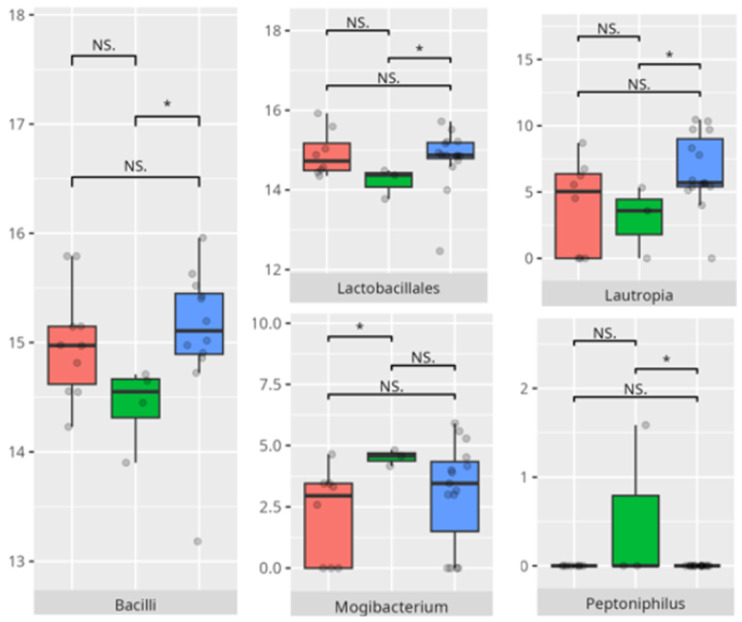
Statistically important differences between groups—daily living skills: domestic skills (red—average, green—low, and blue—high; * *p* ≤ 0.05; NS.—not significant).

The significant differences in the microbiota composition are listed in Table 2 and visualized in Figure 16.

A positive correlation was observed between the domestic skills V-measure and morning salivary cortisol concentrations (r = 0.58), while *Tannerella* abundance was negatively correlated with this measure (r = −0.56) (Table 3).

##### Subdomain: Community Skills

Eleven children demonstrated high or moderately high ability in the community skills subdomain, while 26 exhibited low or moderately low functional scores. Among the higher-performing group, two participants were in the youngest age category, seven were in the middle age, and two were in the oldest age category. FGIDs were reported in six cases, and FS in two. The majority adhered to both protein-based (*n* = 9) and low-calorie (*n* = 9) dietary habits.

In contrast, among those with lower evaluations in community functioning, 10 were from the youngest age group, 14 from the middle, and 2 from the oldest. Twenty-two were boys. FGIDs were noted in 9 cases, while 12 children showed FS. Most followed a protein-rich (*n* = 15) and high-calorie (*n* = 16) diet.

**Figure 17:**
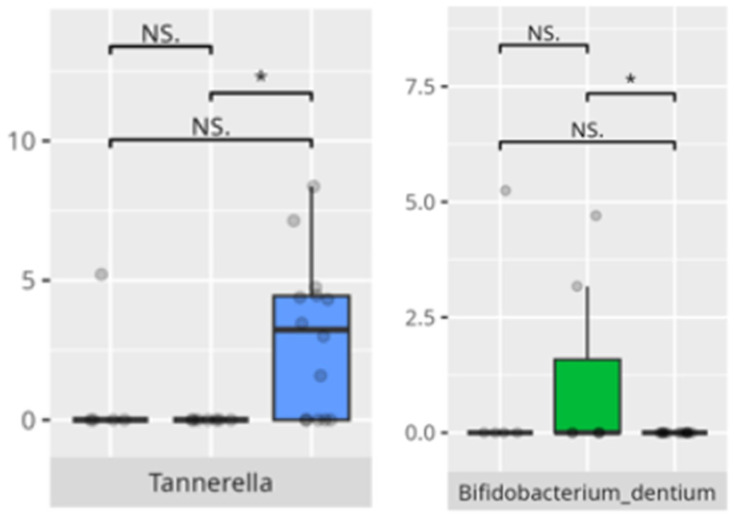
Statistically important differences between groups—daily living skills: community skills (red—average, green—low, and blue—high; * *p* ≤ 0.05; NS.—not significant).

The significant differences in the microbiota composition are listed in Table 2 and visualized in Figure 17.

#### 3.5.3. Domain: Socialization

In the overall socialization domain, 30 children exhibited low or moderately low performance, 11 demonstrated average abilities, and 4 attained high or moderately high competency.

**Figure 18:**
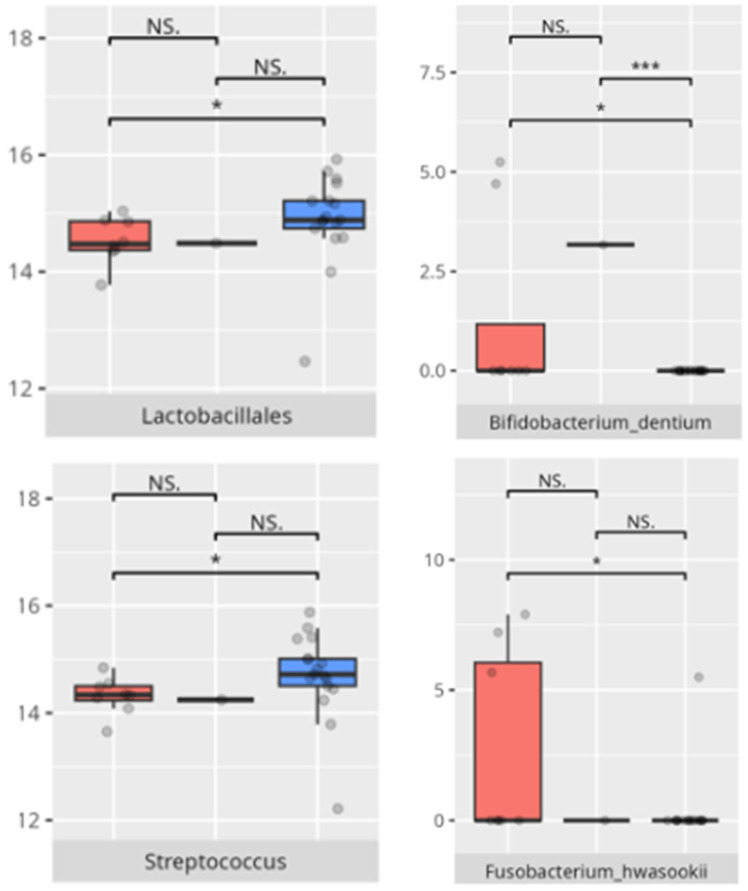
Statistically important differences between groups—socialization: total (red—average, green—low, and blue—high; *—*p* ≤ 0.05, ***—*p* ≤ 0.001; NS.—not significant).

The significant differences in the microbiota composition are listed in Table 2 and visualized in Figure 18.

##### Subdomain: Interpersonal Skills

Six participants demonstrated high or moderately high interpersonal competence, while 28 exhibited low or moderately low functioning. All high-performing children were boys: one from the youngest group, three from the middle group, and two from the oldest group. Three patients presented with FGIDs, and two with FS. Most adhered to a protein-rich (*n* = 3) and low-calorie (*n* = 5) dietary regimen. Within the lower-functioning group, 23 were boys. A total of 11 belonged to the youngest age group, 14 to the middle, and 3 to the oldest. Ten had diagnoses other than childhood autism. FGIDs and FS were reported in 12 children each. The majority followed a protein-based (*n* = 19) and high-calorie (*n* = 16) diet.

**Figure 19:**
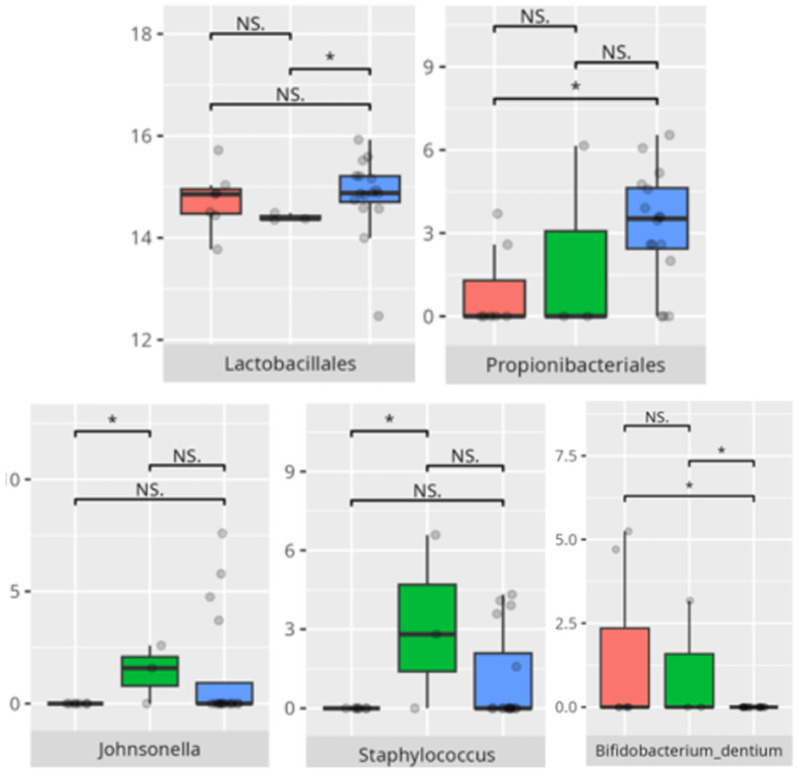
Statistically important differences between groups—socialization: interpersonal skills (red—average, green—low, and blue—high; * *p* ≤ 0.05; NS.—not significant).

The significant differences in the microbiota composition are listed in Table 2 and visualized in Figure 19.

##### Subdomain: Play and Leisure Skills

Five male participants achieved high or moderately high proficiency in the play and leisure skills subdomain, whereas 30 children (25 boys and 5 girls) exhibited low or moderately low functioning. Three high performers were from the middle-aged group, and two were from the oldest. One patient had an FGID, and one exhibited FS. All adhered to a protein-based LCD. Among children with reduced functioning, 11 were in the youngest group, 16 in the middle, and 3 in the oldest. Ten carried diagnoses other than childhood autism. FGIDs and FS were each reported in 13 cases. Most children followed a protein-based (*n* = 19) and high-calorie (*n* = 17) nutritional regimen.

**Figure 20:**
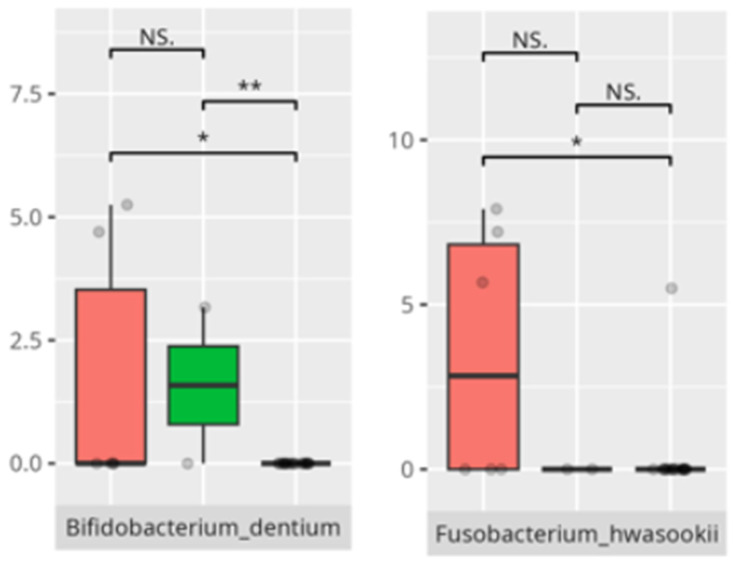
Statistically important differences between groups—socialization: play and leisure (red—average, green—low, and blue—high; *—*p* ≤ 0.05, **—*p* ≤ 0.01; NS.—not significant).

The significant differences in the microbiota composition are listed in Table 2 and visualized in Figure 20.

##### Subdomain: Coping Skills

Four participants attained high or moderately high capacity in coping skills. All of them were boys from either the middle (*n* = 2) or the oldest (*n* = 2) age group. Two experienced FGIDs, and one presented with FS. All followed a PBD, and three consumed an LCD. In the group with diminished coping ability (*n* = 30), 10 were from the youngest age group, 17 from the middle, and 3 from the oldest. A total of 24 were boys, and 19 had childhood autism. FGIDs and FS were noted in 13 and 12 children, respectively. Most adhered to a protein-based (*n* = 21) and low-calorie (*n* = 17) diet.

**Figure 21:**
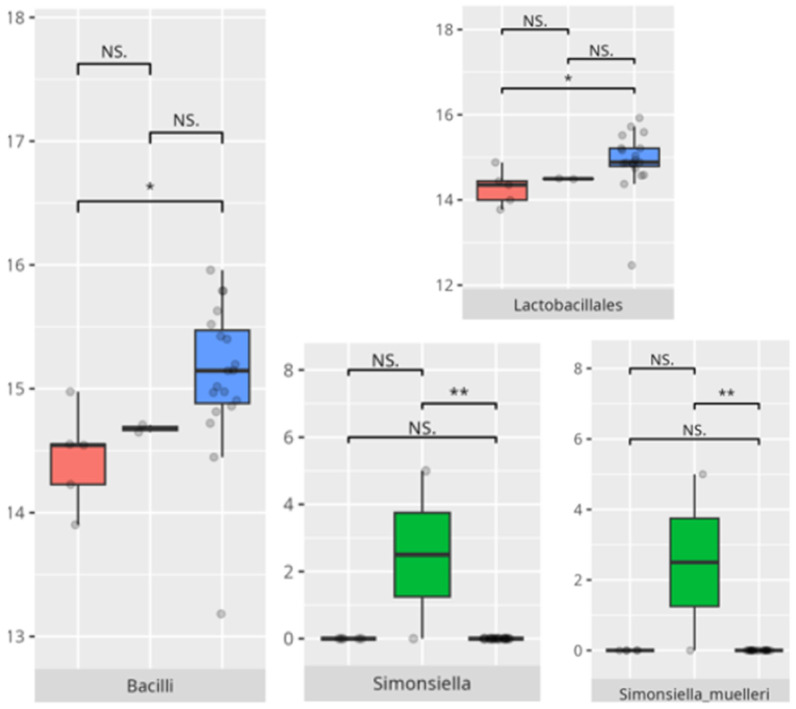
Statistically important differences between groups—socialization: coping skills (red—average, green—low, and blue—high; *—*p* ≤ 0.05, **—*p* ≤ 0.01; NS.—not significant).

The significant differences in the microbiota composition are listed in Table 2 and visualized in Figure 21.

#### 3.5.4. Domain: Motor Skills

Thirteen children demonstrated low or moderately low performance in the motor skills domain. Ten participants exhibited average outcomes, while thirteen achieved high or moderately high results. In accordance with the VABS criteria, children aged 10 years or older were not evaluated in this domain.

**Figure 22:**
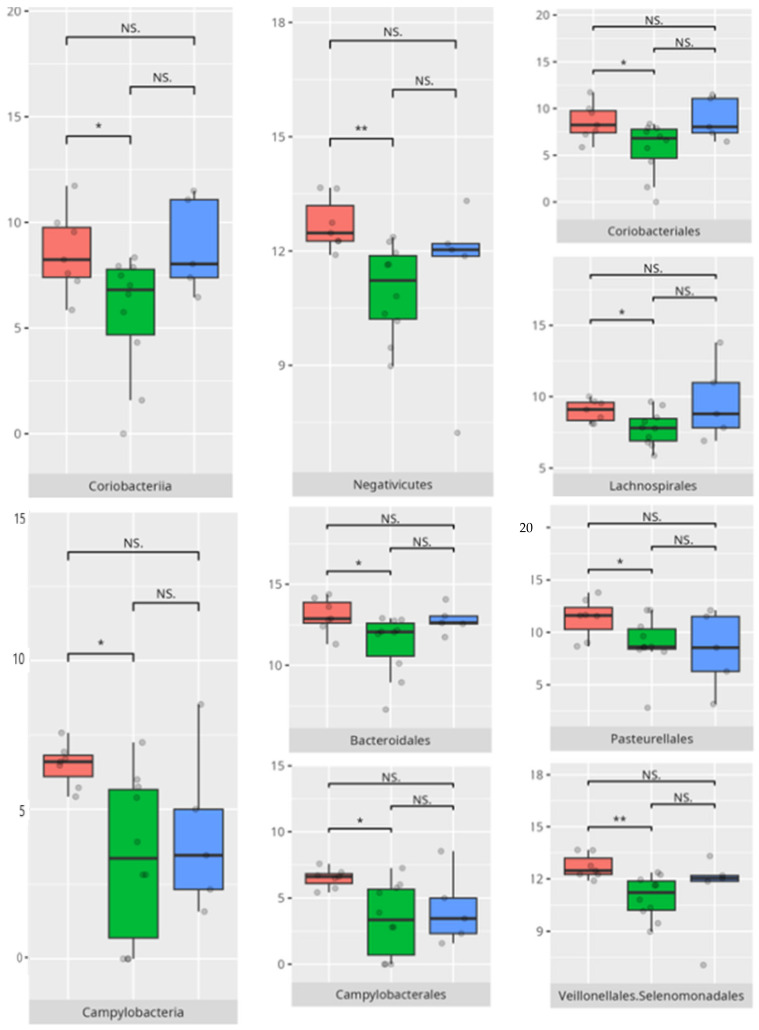

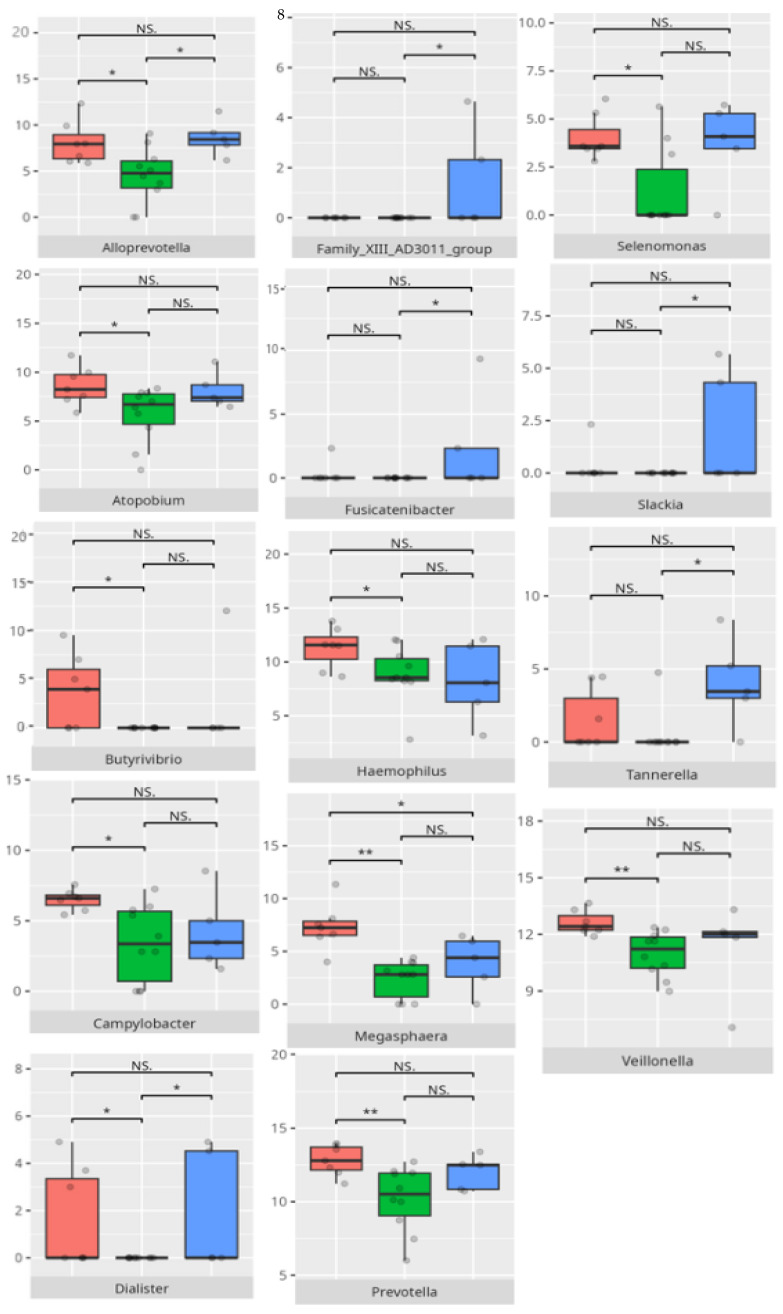
Statistically important differences between groups—motor skills: total (red—average, green—low, and blue—high; *—*p* ≤ 0.05, **—*p* ≤ 0.01; NS.—not significant).

The significant differences in the microbiota composition are listed in Table 2 and visualized in Figure 22.

##### Subdomain: Large Muscle Skills

A total of 17 children (14 boys, 3 girls) demonstrated high or moderately high ability in the large muscle skills subdomain, while 11 (8 boys, 3 girls) exhibited low or moderately low performance. Among the higher-rate group, six were younger children and eleven belonged to the middle-aged group. Seven patients had FGIDs, and seven exhibited FS. Ten followed a PBD, and thirteen consumed an HCD.

In the lower-performing group, six were from the younger age group and five from the middle group. Two presented with FGIDs, and five displayed FS. Most followed a protein-based (*n* = 8) and high-calorie (*n* = 7) diet.

**Figure 23:**
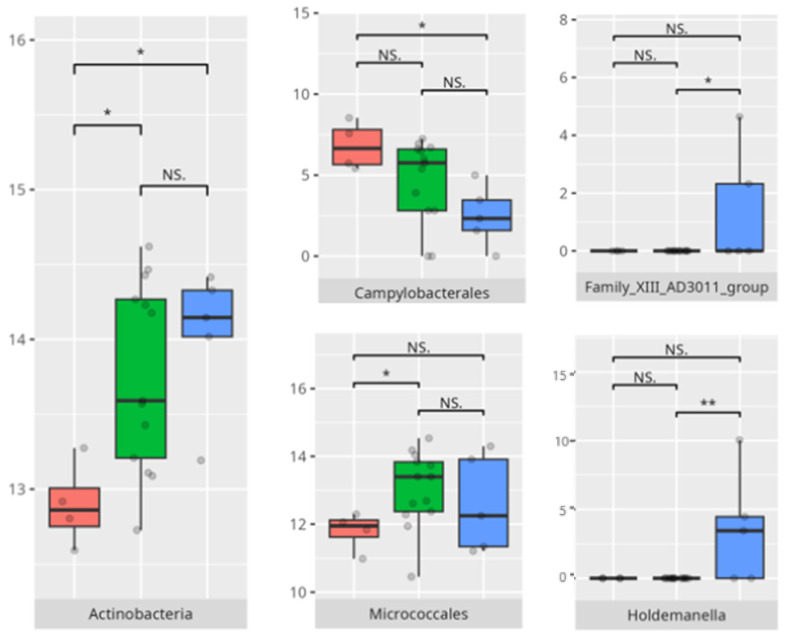

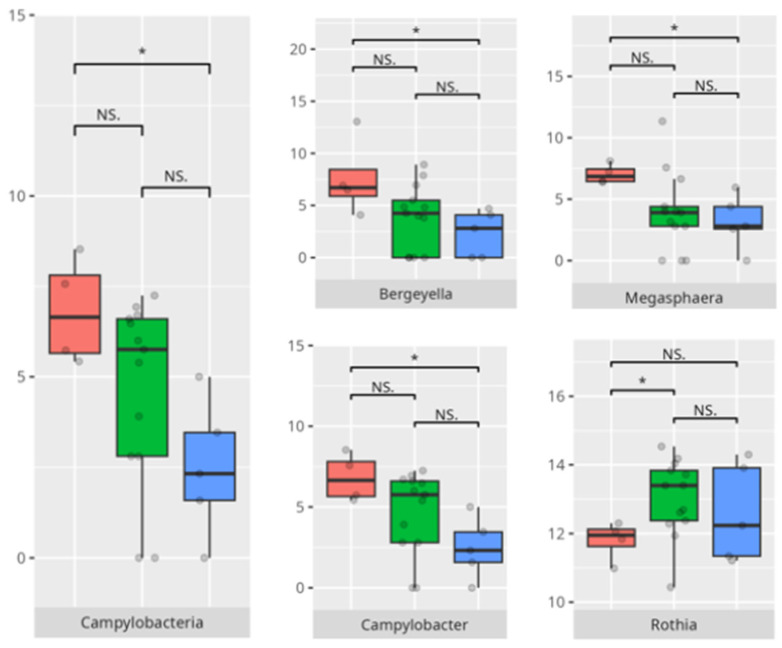
Statistically important differences between groups—motor skills: large muscle skills (red—average, green—low, and blue—high; *—*p* ≤ 0.05, **—*p* ≤ 0.01; NS.—not significant).

The significant differences in the microbiota composition are listed in Table 2 and visualized in Figure 23.

A significant negative correlation was found between the V-rating in large muscle skills and the abundance of Eubacterium brachy (r = −0.59) (Table 3).

##### Subdomain: Small Muscle Skills

Eight children were classified as having high or moderately high capability in the small muscle skills subdomain, while twenty were identified with low or moderately low proficiency. Among the higher-performing group, one child was in the youngest age category and seven were in the middle age group; six were boys. Two presented with FGIDs, and three exhibited FS. Seven followed a PBD, and four adhered to an LCD.

Within the group demonstrating lower performance, eight were younger children and twelve were in the middle age group. Sixteen were boys, and fourteen were diagnosed with childhood autism. FGIDs were reported in six participants, and nine displayed FS. Eleven followed a PBD, and eleven consumed an HCD.

**Figure 24:**
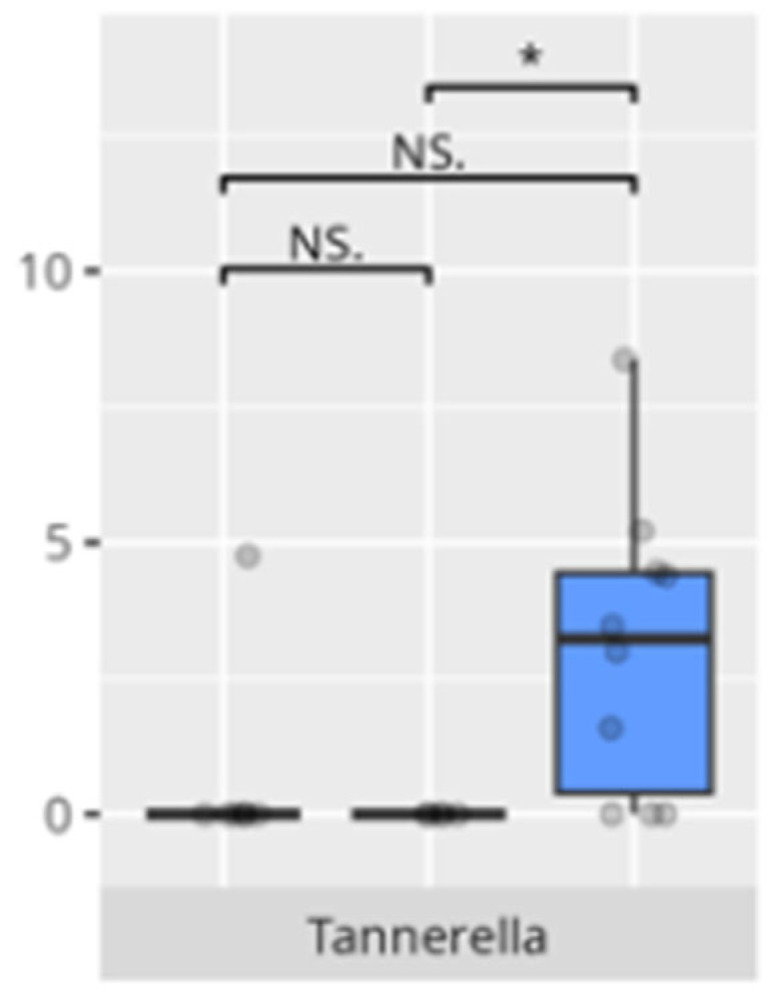
Statistically important differences between groups—motor skills: small muscle skills (red—average, green—low, and blue—high; * *p* ≤ 0.05; NS.—not significant).

The significant differences in the microbiota composition are listed in Table 2 and visualized in Figure 24.

### 3.6. Cortisol Diurnal Release Pattern

All participants exhibited abnormal diurnal cortisol release patterns. Thirteen children displayed a flattened circadian curve, while the remaining 32 exhibited elevated evening salivary cortisol concentrations relative to both morning and afternoon levels.

Children with a flattened cortisol profile were primarily boys in the middle age group, most of whom were diagnosed with childhood autism. Half of this subgroup presented with FGIDs, predominantly functional diarrhea, and a similar proportion exhibited food selectivity. In terms of functional domains, 69% had low performance in communication (*n* = 9), 62% in daily living skills (*n* = 8), and 69% in socialization (*n* = 9). Of the 11 assessed in the motor skills domain, 4 (36%) had diminished performance, and 4 (36%) demonstrated average functional levels.

Children with elevated evening cortisol levels were predominantly boys from the middle (*n* = 19) and youngest (*n* = 10) age groups. FGIDs, primarily functional constipation, were present in 33% of this group, and 27% showed FS. Regarding adaptive functioning, 65% (*n* = 21) exhibited reduced communication skills, 56% (*n* = 18) had difficulties in daily living skills, and 62% (*n* = 20) scored low in socialization. Among the 24 children evaluated for motor skills, 10 (42%) demonstrated high proficiency.

## 4. Discussion

### 4.1. Oral Microbiota Composition and Population Structure

The present study offers compelling insights into the associations between oral microbiota composition and multiple physiological, dietary, behavioral, and developmental parameters in children with ASD. Through an integrated analysis involving microbiome sequencing, dietary profiling, gastrointestinal symptomatology, adaptive functioning, and cortisol secretion patterns, the findings contribute to a nuanced understanding of the oral–gut–brain axis and its potential implications for neurodevelopmental disorders. These results not only reinforce previously established associations but also offer novel correlations that merit further exploration and could inform therapeutic interventions. The identification of 363 microbial species spanning 11 phyla underscores the oral cavity’s complex ecological network and its potential systemic influence. Notably, the microbial shifts identified across sex, age, and birth mode reflect a convergence of host-related variables shaping microbial colonization. The increased presence of *Lactobacillus salivarius* and *Streptococcus sobrinus* in females aligns with findings suggesting hormonal modulation of oral bacterial profiles [17,18]. Similarly, Cesarean-associated enrichment of *Fusobacterium nucleatum* subsp. *kwasookii* may reflect altered initial colonization pathways, corroborated by neonatal microbiome studies [19]. The microbial divergence in older children, particularly elevated *Capnocytophaga ochracea* and *Corynebacterium striatum*, may indicate age-dependent shifts in immune–microbial interplay or dietary transition effects [20].

This study reveals that in children with ASD, pro-inflammatory bacteria are enriched in Cesarean-born participants, highlighting the role of the mode of delivery in the formation of microbiota composition. Other species with potentially pathogenetic activity were observed in older children, supporting the role of environmental factors. Anti-inflammatory species seem to be more prevalent in female patients with ASD, which can be connected to genetic or hormonal factors.

### 4.2. Functional Gastrointestinal Disorders and Microbiota Profiles

A significant proportion of children fulfilled criteria for FGIDs, paralleling epidemiological estimates in ASD cohorts [6]. Our findings demonstrate distinct oral microbiota signatures associated with specific FGID phenotypes. The enrichment of *Negativicutes* and *Veillonellales/Selenomonadales* in children with functional diarrhea echoes prior gut microbiota studies linking these taxa to pro-inflammatory metabolic pathways [6]. The predominance of *Corynebacteriales* in bloating and constipation parallels their mucosal colonization characteristics and potential roles in biofilm formation. Genera such as *Selenomonas* and *Megasphaera* emerged as discriminant markers for FGIDs, consistent with their anaerobic fermentation profiles and possible production of short-chain fatty acids implicated in gut motility [6,18]. Conversely, *Bacteroides* dominance in asymptomatic individuals may reflect a more stable microbial niche. The species-specific signatures, including *Lactobacillus fermentum* and *Streptococcus anginosus* in diarrheal subjects, may suggest a perturbation in lactic acid production and mucosal immunity.

This study identifies distinct oral microbiota profiles linked to FGIDs in children with ASD, with pro-inflammatory bacteria enriched in those with functional gastrointestinal symptoms, while anti-inflammatory-associated *Bacteroides* predominate in asymptomatic individuals.

### 4.3. Dietary Patterns and Food Selectivity

Despite prevalent high protein intake, no direct associations were observed between protein quantity and specific genera, possibly due to confounding dietary selectivity and food processing. This may reflect increased parental emphasis on protein-rich diets, especially in children exhibiting food selectivity—who often reject vegetables and consume limited fruit—that is typically restricted to a single preferred item. Their diets commonly include highly processed protein sources. This underscores the need for nutrient quality assessment rather than macronutrient totals alone.

The elevated *Enterobacteriales* and *Alloprevotella* in children with FS corroborate previous findings linking selective diets with enrichment of facultative anaerobes and potentially pro-inflammatory taxa [18]. *Bifidobacterium longum*, noted in non-FGID children, is a well-known beneficial species with immunomodulatory functions [18]. Furthermore, the observed negative correlation between complex carbohydrate intake and *Streptococcus mutans* aligns with the latter’s saccharolytic niche and its established role in dental caries pathogenesis. The positive correlation between saturated fat intake and *Mobiluncus curtisii* suggests a diet-dependent selection for lipid-adapted bacterial taxa, warranting further exploration of lipid–microbiome–host interactions. The association between saturated fat intake and the abundance of *Mobiluncus curtisii* is less well documented. While specific studies directly linking saturated fat consumption to *M. curtisii* are limited, research indicates that high-fat diets can influence gut microbiota composition. For instance, a study found that diets high in saturated fats were associated with changes in gut microbiota, including alterations in bacterial taxa related to metabolic disorders [13,21,22].

This study found that children with FS exhibited increased pro-inflammatory bacteria. Additionally, saturated fat intake correlated positively with *Mobiluncus curtisii*, suggesting diet-driven shifts toward lipid-adapted, potentially pro-inflammatory taxa [23].

### 4.4. Functioning and Microbiota Composition

In the domain of communication, microbial disparities paralleled adaptive functioning levels. Children with average communication scores exhibited higher *Propionibacteriales* and *Verrucomicrobiae* abundance, a pattern possibly reflective of oral mucosal immune tolerance states [13]. In contrast, *Catonella* and *Oribacterium* enrichment in high scorers may signify a more regulated microbial environment or dietary variance. Intriguingly, *Akkermansia* and *Eggerthella,* dominating in average performers, are taxa increasingly associated with gut–brain axis modulation and mucin degradation, respectively [19,24].

The observed association between low receptive scores and increased *Actinomyces* and *Tannerella* abundance suggests a potential role of inflammatory-prone taxa in shaping neurobehavioral outcomes. High scorers’ enrichment in *Haemophilus* and *Saccharimonadaceae* is notable, given emerging evidence linking these genera to immunological balance and neurosignaling molecules [18]. Similar trends were evident in the expressive communication domain. Higher scores correlated with *Haemophilus* and *Fusobacterium*—genera often implicated in maintaining oral health—while low-scoring participants showed dominance of *Pseudopropionibacterium* and *Tannerella*, potentially indicating dysbiotic states with systemic repercussions [25,26].

Variations in microbiota associated with writing skills were subtle yet informative. Children with average scores demonstrated increased *Verrucomicrobiae* and *Oscillospirales*, both of which have been linked to mucosal stability and neuroimmune homeostasis [13,21,22]. The elevated abundance of *Bacteroides* in average scorers could imply more diverse and fiber-rich diets, conducive to favorable neurodevelopmental outcomes [27]. Conversely, *Lautropia* in low performers has been previously associated with oral dysbiosis and early dental disease risk. The researchers suggest that an overrepresentation of *Lautropia* may serve as a marker for oral microbial imbalance, potentially impacting overall oral health [27].

In this study, elevated coping abilities were exclusively observed in boys from the older age categories, with a predominance of protein-based and LCDs. These dietary characteristics may suggest a more regulated metabolic environment, potentially linked to reduced microbial diversity but greater compositional stability. Participants with lower coping skills demonstrated an increased abundance of *Bacilli* and *Lactobacillales*, taxa frequently associated with gastrointestinal disturbances and mucosal inflammation [28]. The enrichment of *Simonsiella* in this group further supports a state of oral dysbiosis, as this genus has been implicated in disrupted microbial homeostasis [29].

Children with average motor skills exhibited a more diverse microbial profile than their high-performing peers, with notable increases in *Coriobacteria, Campylobacteria*, and *Negativicutes*. These findings might reflect a transitional microbiota associated with systemic inflammation or metabolic inefficiency [30]. The increased prevalence of *Campylobacteriales* and *Veillonellales/Selenomonadales* aligns with previous work linking these taxa to motor and cognitive impairments in patients with Parkinson’s disease [31,32]. At the genus level, the enrichment of *Prevotella* and *Megasphaera* among average performers, as opposed to high scorers, could indicate a dietary influence. Some studies found an association between enrichment of the *Prevotella* genus and a diet high in carbohydrates and fiber. For example, a review of the literature made by Precup indicates that *Prevotella* species are correlated with diets rich in plants, rich in carbohydrates, and fiber [33]. Similarly, studies have shown that a diet high in protein and animal fats is associated with a predominance of bacteria from the *Bacteroides* genus, while a diet high in carbohydrates and fiber favors the dominance of *Prevotella*, possibly tied to fiber or carbohydrate intake [34].

In the large muscle skills subdomain, performance correlated with nuanced microbial shifts. High performers demonstrated greater levels of *Rothia* and *Micrococcales*, genera often associated with oral health and environmental stability [35]. Increased abundance of *Actinobacteria* and *Campylobacteriales*—including genera like *Actinomyces* and *Campylobacter*—has been associated with pro-inflammatory states and gut dysbiosis. These taxa have been linked to inflammatory bowel diseases (IBDs), where they may exacerbate inflammation and disease progression. Similarly, *Holdemanella* has been implicated in inflammatory responses, though research on this genus is still emerging [36]. The negative correlation between *Eubacterium brachy* and performance in this subdomain (r = −0.59) suggests a potentially deleterious role of this taxon in neuromotor regulation [37].

Children with reduced fine motor skills showed a clear enrichment of *Tannerella*, a known periodontal pathogen associated with oral inflammation and systemic repercussions. There is no data regarding the relationship between the identified pathogen and children’s development.

This study found that pro-inflammatory bacteria such as *Actinomyces*, *Tannerella*, *Campylobacteriales*, and *Veillonellales* were enriched in children with lower communication, motor, and coping skills, suggesting a link between inflammatory-prone oral microbiota and neurobehavioral challenges. Conversely, anti-inflammatory or health-associated taxa like *Propionibacteriales*, *Verrucomicrobiae*, *Haemophilus*, *Rothia*, and *Bacteroides* correlated with higher adaptive functioning and better neurodevelopmental outcomes, highlighting the potential role of oral microbial balance in communication and motor skills in children.

### 4.5. Cortisol Diurnal Release Pattern

All participants exhibited abnormal diurnal cortisol patterns, characterized by two primary subtypes: flattened curves and elevated evening peaks. The flattened cortisol profile was associated with reduced adaptive performance across multiple domains and was more prevalent among children diagnosed with ASD and functional diarrhea. This pattern is consistent with prior studies linking hypothalamic–pituitary–adrenal axis dysregulation to impaired stress resilience and gastrointestinal dysfunction [38,39]. Conversely, elevated evening cortisol levels were observed in participants with enhanced motor skill performance, yet co-occurred with deficits in social and communication domains, suggesting a complex and potentially compensatory neuroendocrine response. These divergent cortisol signatures, together with variations in gut microbiome composition, may define distinct biobehavioral phenotypes within the autism spectrum. Collectively, these findings indicate that alterations in cortisol rhythms and microbial ecology are associated with heterogeneous neurodevelopmental profiles in children with ASD. A nuanced understanding of these biobehavioral phenotypes could inform targeted interventions addressing both endocrine and microbial factors to enhance adaptive functioning [38,39,40,41].

### 4.6. Limitations

This study provides novel insights into the complex interplay between oral microbiota and behavioral, dietary, gastrointestinal, and neuroendocrine parameters in children with ASD. Nevertheless, several limitations should be acknowledged. First, the cross-sectional design precludes any inference of causality between microbiota composition and clinical phenotypes. Longitudinal studies are needed to determine whether observed microbial patterns are a cause or consequence of ASD-related symptoms. Second, although the cohort was rigorously selected, the sample size remains relatively small, particularly for subgroup analyses involving sex, age, diet, or adaptive functioning domains. This may limit the generalizability of the findings and underscores the need for replication in larger, more diverse populations.

## 5. Conclusions

This study highlights intricate, multidimensional associations between the oral microbiota and behavioral, gastrointestinal, and nutritional parameters in children with ASD. These findings reinforce the hypothesis of a bidirectional microbiota–gut–brain axis, with the oral ecosystem acting as a potential upstream modulator. Longitudinal studies integrating metatranscriptomic, metabolomic, and neuroimaging data are warranted to delineate causal pathways and therapeutic implications.

## Figures and Tables

**Table 1:** Participants’ characteristics.

**Total Number of Participants**	**45**
**Sex**	
Girl	7
Boy	38
**Age**	7.9 ± 3.35
2–6 years	12
6–14 years	28
15–18 years	5
**Type of delivery**	
Vaginal delivery	31
Cesarean section	14
**Gastrointestinal symptoms**	
Functional constipation	8
Functional diarrhea	9
Functional bloating	7
Food selectivity	16
**Diet**	
Average daily protein intake (g/day)	90.05 ± 31.06
Protein intake per kilogram of body weight (g/kg BW)	3.81 ± 1.71
Average daily carbohydrate intake (g/day)	251.02 ± 62.66
Percentage of energy from carbohydrates (%)	51.41 ± 9.31
Proportion of simple carbohydrates in total carbohydrate intake (%)	41.31± 7.82
Average daily fat intake (g/day)	79.51 ± 31.32
Average fat intake per kilogram of body weight (g/kg BW)	3.3 ± 1.7
Percentage of unsaturated fats in total fat intake (%)	59.89 ± 5.30

**Table 2:** Comparison between patients’ groups in terms of microbiota composition.

Feature	Bacteria	*p*-Value≤
**Class**		
Age: younger children	*Alphaproteobacteria*	0.05
Other diagnosis	*Fusobacteria*	0.05
	*Saccharimonadia*	0.05
Diarrhea	*Negativicutes*	0.05
Communication—total (average over low)	*Verrucomicrobiae*	0.05
Communication—total (average above high)	*Bacilli*	0.05
Receptive communication (average over high)	*Actinobacteria*	0.05
Expressive communication (low over high)	*Bacilli*	0.05
Expressive communication (average over high)	*Actinobacteria*	0.05
Communication—writing skills (average over low)	*Verrucomicrobiae*	0.01
Daily living skills—total (low over high)	*Bacilli*	0.05
Daily living skills—personal skills (low over high)	*Bacilli*	0.05
Daily living skills—domestic skills (low over high)	*Bacilli*	0.05
Socialization—coping skills (low over average)	*Bacilli*	0.05
Socialization—coping skills (low over high)	*Bacilli*	0.05
Motor skills—total (average over high)	*Coriobacteria*	0.05
	*Campylobacteria*	0.05
	*Negativicutes*	0.01
Motor skills—using large muscles (low over average)	*Actinobacteria*	0.05
Motor skills—using large muscles (average over low)	*Campylobacteria*	0.05
Motor skills—using large muscles (high over average)	*Actinobacteria*	0.05
**Order**		
Age (younger over school age)	*Actinomycetales*	0.05
	*Propionibacteriales*	0.05
Age (teenagers over school age)	*Pseudomonadales*	0.01
Other diagnosis	*Fusobacteriales*	0.05
	*Propionibacteriales*	0.05
	*Saccharimonadales*	0.05
Low protein diet	*Pseudomonadales*	0.05
Food selectivity	*Enterobacteriales*	0.05
Diarrhea	*Veillonellales Selenomonadales*	0.05
Bloating	*Corynebacteriales*	0.05
Constipation	*Corynebacteriales*	0.05
Communication—total (low over high)	*Actinomycetales*	0.05
Communication—total (average over low)	*Verrucomicrobiales*	0.05
Communication—total (average above high)	*Propionibacteriales*	0.05
Expressive communication (low over high)	*Actinomycetales*	0.05
	*Propionibacteriales*	0.05
Receptive communication (low over high)	*Actinomycetales*	0.05
	*Propionibacteriales*	0.05
Communication—writing skills (average over low)	*Verrucomicrobiales*	0.01
Communication—writing skills (average over high)	*Oscillospirales*	0.05
Daily living skills—total (low over high)	*Lactobacillales*	0.05
Daily living skills—personal skills (low over high)	*Lactobacillales*	0.05
	*Propionibacteriales*	0.05
Daily living skills—personal skills (average over high)	*Propionibacteriales*	0.05
Daily living skills—domestic skills (low over high)	*Lactobacillales*	0.05
Socialization—total (low over average + high)	*Lactobacillales*	0.05
Socialization—interpersonal adaptive level (low over average)	*Propionibacteriales*	0.05
Socialization—interpersonal adaptive level (low over high)	*Lactobacillales*	0.05
Socialization—coping skills (low over average)	*Lactobacillales*	0.05
Motor skills—total (average over high)	*Bacteroidales*	0.05
	*Campylobacteriales*	0.05
	*Coriobacteriales*	0.05
	*Lachnospirales*	0.05
	*Pasteurellales*	0.05
	*Veillonellales Selenomonadales*	0.01
Motor skills—using large muscles (average over high)	*Campylobacteriales*	0.05
Motor skills—using large muscles (high over average)	*Micrococcales*	0.05
**Genus**		
Sex: female	*Faecalibacterium*	0.05
Age (younger over school age)	*Actinomyces*	0.05
	*Microbacterium*	0.05
	*Pseudopropionibacterium*	0.05
Age (teenagers over younger)	*Candidatus saccharibacteria*	0.05
Age (school age over teenagers)	*Candidatus saccharibacteria*	0.05
	*Pseudomonas*	0.05
Vaginal delivery	*Butyrivibrio*	0.05
	*Prevotella*	0.05
	*Veillonella*	0.05
Other diagnosis	*Holdemanella*	0.05
	*Leprotrichia*	0.01
	*Pseudopropionibacterium*	0.01
Low protein diet	*Finapoldia*	0.05
	*Tannerella*	0.05
Food selectivity	*Alloprevotella*	0.05
	*Family XII AD3011*	0.05
	*Peptoclostridium*	0.05
No FGIDs	*Bacteroides*	0.05
FGIDs	*Selenomonas*	0.05
No diarrhea	*Actinobacillus*	0.05
Diarrhea	*Megasphaera*	0.05
	*Prevotella*	0.05
	*Selenomonas*	0.01
	*Veillonella*	0.05
Bloating	*Bergeyella*	0.05
	*Corynebacterium*	0.05
Constipation	*Bergeyella*	0.05
	*Corynebacterium*	0.05
	*Eikenella*	0.05
	*Actinomyces F0332*	0.05
Expressive communication (high over average)	*Haemophilus*	0.05
	*Peptococcus*	0.05
	*Steptobacillus*	0.05
	*Fusobacterium*	0.05
Communication—total (low over high)	*Tannerella*	0.05
Communication—total (average over low)	*Akkermansia*	0.05
	*Eggerthella*	0.05
	*Granullicatella*	0.05
Communication—total (average above high)	*Pseudopropionibacterium*	0.05
Communication—total (high over average)	*Catonella*	0.05
	*Oribacterium*	0.05
Receptive communication (low over high)	*Actinomyces*	0.05
	*Pseudopropionibacterium*	0.05
	*Tannerella*	0.05
Receptive communication (average over low)	*Alloscardovia*	0.05
Receptive communication (high over average)	*Haemophilus*	0.05
	*Saccharimonadaceae*	0.05
Expressive communication (low over average)	*Peptostreptococcus*	0.05
	*Saccharimonadaceae*	0.05
	*Tannerella*	0.05
	*Fusobacterium*	0.05
Expressive communication (low over high)	*Pseudopropionibacterium*	0.05
	*Selemonas*	0.05
	*Tannerella*	0.05
Expressive communication (average over high)	*Kingella*	0.05
	*Selenomonas*	0.05
Communication—writing skills (low over high)	*Lautropia*	0.05
Communication—writing skills (average over low)	*Akkermansia*	0.01
	*Bacteroides*	0.05
	*Eggerthella*	0.01
Communication—writing skills (average over high)	*Bacteroides*	0.05
Daily living skill total (low over average)	*Tannerella*	0.05
Daily living skill total (average over high)	*Abiotrophia*	0.05
Daily living skills—personal skills (low over average)	*Tannerella*	0.05
Daily living skills—personal skills (low over high)	*Pseudopropionibacterium*	0.05
	*Tannerella*	0.05
Daily living skills—personal skills (average over high)	*Abiotrophia*	0.05
	*Pseudopropionibacterium*	0.05
Daily living skills—domestic skills (low over high)	*Lautropia*	0.05
Daily living skills—domestic skills (high over low)	*Peptoniphilus*	0.05
Daily living skills—domestic skills (high over average)	*Mogibacterium*	0.05
Daily living skills—community skills (low over high)	*Tannerella*	0.05
Socialization—total (low over average)	*Streptococcus*	0.05
Socialization—interpersonal skills (high over average)	*Johnsonella*	0.05
	*Staphylococcus*	0.05
Socialization—coping skills (high over low)	*Simonsiella*	0.01
	*uncultured*	0.05
Motor skills—total (low over high)	*Alloprevotella*	0.05
	*Eubacterium brachy*	0.05
	*Dialister*	0.05
	*Family XIII AD3011*	0.05
	*Fusicatenibacter*	0.05
	*Slackia*	0.05
	*Tannerella*	0.05
Motor skills—total (average over low)	*Megasphaera*	0.05
Motor skills—total (average over high)	*Alloprevotella*	0.05
	*Atopobium*	0.05
	*Butyrivibrio*	0.05
	*Campylobacter*	0.05
	*Dialister*	0.05
	*Haemophilus*	0.05
	*Megasphaera*	0.01
	*Prevotella*	0.01
	*Selenomonales*	0.05
	*Veillonella*	0.01
Motor skills—using large muscles (low over high)	*Family XIII AD3011 group*	0.05
	*Holdemanella*	0.01
Motor skills—using large muscles (average over high)	*Bergeyella*	0.05
	*Campylobacter*	0.05
	*Megasphaera*	0.05
Motor skills—using large muscles (high over average)	*Rothia*	0.05
Motor skills—using small muscles (low over high)	*Tannerella*	0.05
**Species**		
Sex: female	*Lactobacillus salivarius*	0.01
	*Steptococcus sobrinus*	0.05
Age (teenagers over school age)	*Capnocytophaga ochacea*	0.01
	*Corynebacterium strictum*	0.05
Delivery: Cesarean section	*Fusobacterium kwasookii*	0.05
Food selectivity	*Lactobacillus fermentum*	0.05
No FGIDs	*Bifidobacterium longum*	0.05
Diarrhea	*Lactobacillus fermentum*	0.05
	*Streptococcus anginosus*	0.05
Communication—total (average over low)	*Lactobacillus reuteri*	0.05
Daily living skill total (high over low)	*Bifidobacterium dentium*	0.05
Daily living skills—community skills (high over low)	*Bifidobacterium dentium*	0.05
Socialization—total (low over high)	*Bifidobacterium dentium*	0.001
Socialization—total (average over low)	*Bifidobacterium dentium*	0.05
	*Fusobacterium hwasookii*	0.05
Socialization—interpersonal (average over high)	*Bifidobacterium dentium*	0.05
Socialization—interpersonal (high over low)	*Bifidobacterium dentium*	0.05
Socialization—coping skills (high over low)	*Simonsiella muelleri*	0.01
Socialization—play and leisure (average over low)	*Bifidobacterium dentium*	0.05
	*Fusobacterium hwasookii*	0.05
Socialization—play and leisure (high over low)	*Bifidobacterium dentium*	0.01

FGIDs—functional gastrointestinal disorders.

**Table 3:** List of correlations between different features and bacteria abundance or cortisol level.

Feature	Bacteria	Correlation (r)
**Genus**		
Age	*Eubacterium brachy group*	0.62
Weight	*Eubacterium brachy group*	0.53
Fat saturated (mean)/fat total (mean)	*Alloscardoria*	0.69
	*Butyryvibrio*	0.57
	*Mobilincus*	0.81
Liquids (mean)	*Eubacterium yurii*	0.55
Using large muscles (v-score)	*Eubacterium brachy group*	−0.59
Daily living skills—domestic (v-score)	morning cortisol	0.58
	*Tannerella*	−0.56
Daily living skills—personal (v-score)	*Tannerella*	−0.53
**Species**		
Fat saturated (mean)/fat total (mean)	*Bifidobacterium dentium*	0.54
	*Mobilincus curtisii*	0.81
kcal from carbohydrates/total carbohydrates (mean)	*Streptococcus mutans*	−0.53
Complex carbohydrates (mean)	*Streptococcus mutans*	−0.58

Statistical methods used for data in Table 3: Pearson correlation coefficient used for all correlations except for mean complex carbohydrates, mean liquids, morning cortisol level, age, and weight, where Spearman’s rank correlation coefficient was calculated.

## Data Availability

The raw data supporting the conclusions of this article will be made available by the authors upon request.

## Abbreviations

The following abbreviations are used in this manuscript:

ASD	Autism Spectrum Disorder
ChBD	Carbohydrate-Based Diet
ELISA	Enzyme-Linked Immunosorbent Assay
FGID	Functional Gastrointestinal Disorder
FS	Food Selectivity
HCD	High-Calorie Diet
ICD-10	International Statistical Classification of Diseases and Related Health Problems—10th Revision
LCD	Low-Calorie Diet
PBD	Protein-Based Diet
VABS	Vineland Adaptive Behavior Scale
